# Traditional Knowledge and Biocultural Roles of Edible Flowers in Local Food Systems of Baise City, Guangxi, China

**DOI:** 10.3390/biology15110873

**Published:** 2026-06-01

**Authors:** Wei Shen, Xiangtao Cen, Zisong Wang, Piyaporn Saensouk, Surapon Saensouk, Auemporn Junsongduang, Pathomthat Srisuk, Khwanjai Thanakornjuk, Tammanoon Jitpromma

**Affiliations:** 1 Agriculture and Food Engineering College, Baise University, Baise 533000, China; bsxyshenwei@bsuc.cn (W.S.); nxycenxiangtao@bsuc.cn (X.C.); wangzis@bsuc.cn (Z.W.); 2 Diversity of Family Zingiberaceae and Vascular Plant for Its Applications Research Unit, Mahasarakham University, Maha Sarakham 44150, Thailand; pcornukaempferia@yahoo.com (P.S.); jitpromma.t@gmail.com (T.J.); 3 Walai Rukhavej Botanical Research Institute, Mahasarakham University, Maha Sarakham 44150, Thailand; 4 Department of Biology, Faculty of Science, Mahasarakham University, Maha Sarakham 44150, Thailand; 5 Program of Biology, Department of Science and Technology, Faculty of Liberal of Art and Science, Roi Et Rajabhat University, Roi Et 45120, Thailand; a.junsongduang@reru.ac.th; 6 Department of Pharmaceutical Technology, Faculty of Pharmaceutical Sciences, Khon Kaen University, Khon Kaen 40002, Thailand; spatho@kku.ac.th; 7 Tha Uthen Hospital, 23/23 Moo.6 Nontan Thauthen, Nakhonphanom 48120, Thailand; tkjai2521@yahoo.co.th

**Keywords:** China, edible flowers, edible plants, ethnobotany, ethnomedicine, medicinal plants

## Abstract

Edible flowers are important components of traditional food systems and biocultural practices in southern China, yet their ethnobotanical significance remains poorly documented. This study investigated the diversity, traditional uses, and cultural importance of edible flowers in Baise City through semi-structured interviews, market surveys, and field observations with local informants. Quantitative ethnobotanical indices, including the Cultural Food Significance Index (CFSI), Fidelity Level (FL), and Informant Consensus Factor (ICF), were applied to evaluate cultural and medicinal importance. A total of 96 edible flower taxa belonging to 77 genera and 44 families were documented. Most species were native to China, herbaceous in growth form, and collected from wild habitats. Inflorescences were the most commonly utilized floral organs. Edible flowers were used as vegetables, herbal teas, medicinal edible plants, natural food colorants, condiments, desserts, and snack foods. Species such as *Emilia sonchifolia* (L.) DC., *Plantago asiatica* L., and *Solanum americanum* Mill. showed high cultural significance. A total of 64 taxa were recognized as medicinal edible plants, and high ICF values indicated strong agreement among informants regarding ethnomedicinal uses. These findings demonstrate the important roles of edible flowers in local food systems, traditional healthcare, and biocultural heritage, emphasizing their relevance for biodiversity conservation and sustainable food practices.

## Simple Summary

Edible flowers have long been used as foods, beverages, and traditional medicines in many parts of China, but information about their diversity and cultural importance is still limited in southern China. This study documented traditional knowledge related to edible flowers in Baise City through interviews, market surveys, and field observations. A total of 96 edible flower species were recorded, including plants used as vegetables, herbal teas, natural food colorants, condiments, desserts, and medicinal foods. Many species were collected from wild habitats and played important roles in daily diets and local healthcare practices. Several edible flowers were highly valued by local communities because of their frequent use, medicinal properties, and cultural importance. The study also showed strong agreement among local people regarding the medicinal uses of many species, especially for treating common illnesses such as colds, sore throat, inflammation, and heat-related conditions. These findings highlight the close relationship between food, traditional medicine, and cultural heritage in local communities. The documentation of edible flower knowledge is important for preserving traditional practices, supporting biodiversity conservation, and promoting sustainable local food systems for future generations.

## 1. Introduction

Throughout human history, people have developed intricate relationships with plants, which serve as essential resources for food, medicine, and cultural practices [1]. Among these, food plants represent a fundamental component of biocultural diversity, linking ecological systems with human societies and cultural traditions [2]. Edible plants not only contribute to nutrition and food security but also reflect accumulated traditional knowledge and adaptive strategies shaped by local environments [3]. Within this broad category, edible flowers constitute a unique and culturally significant group of plant resources [4].

Edible flowers (EFs) are generally defined as flowers or floral parts that are consumed as food, either directly or as ingredients in culinary preparations. In a broader ethnobotanical context, the term “edible flowers” also includes inflorescences and composite floral structures, as many species are consumed in their aggregated floral form rather than as single, isolated flowers. They are used in a variety of ways, including vegetables, beverages, condiments, and decorative elements in traditional dishes [5]. In recent years, edible flowers have attracted increasing attention due to their nutritional value and functional properties. Many species are rich in bioactive compounds such as flavonoids, phenolics, vitamins, and essential minerals, which contribute to their antioxidant and health-promoting effects [6,7]. Consequently, edible flowers are increasingly recognized not only as traditional food resources but also as functional foods with potential applications in modern diets and nutraceutical development [8].

Despite growing interest in their nutritional and phytochemical properties, ethnobotanical studies on edible flowers remain limited in many regions. Traditional knowledge associated with edible flowers encompasses species selection, harvesting practices, seasonal availability, preparation techniques, and cultural meanings. This knowledge is often transmitted orally within communities and is closely tied to local ecological conditions and cultural identities [9,10]. However, rapid socio-economic changes, urbanization, and shifts in dietary preferences—particularly among younger generations—are leading to the gradual erosion of this knowledge [11]. As a result, documenting and preserving traditional knowledge related to edible flowers has become increasingly important [12].

China has a long history of utilizing flowers as food, with cultural records dating back thousands of years. Historical literature and classical texts reflect the deep-rooted tradition of flower consumption, highlighting its esthetic, nutritional, and symbolic values [13,14,15]. In many regions, especially those characterized by high biodiversity and cultural diversity, edible flowers remain an integral part of local diets and traditional practices [16]. South China, in particular, is recognized for its rich floristic diversity and the extensive use of wild and cultivated plants by various ethnic groups [17,18].

Baise City, located in the northwestern part of Guangxi Zhuang Autonomous Region, is characterized by diverse ecological landscapes, including karst formations, forests, and agricultural systems. The region is inhabited by multiple ethnic communities, each possessing distinct cultural traditions and extensive knowledge of plant use [19]. These environmental and cultural conditions contribute to a rich diversity of edible plant resources, including numerous edible flower species. Local communities have long utilized these flowers for food, beverages, and traditional dishes, reflecting their adaptation to the surrounding environment and the integration of ecological knowledge into daily life [20].

Although ethnobotanical studies on EFs have been conducted in several regions of China, particularly in areas characterized by high biological and cultural diversity, most previous research has focused on documenting species diversity, traditional uses, nutritional composition, and phytochemical properties [4,21]. Nevertheless, ethnobotanical investigations of edible flowers remain limited in many local regions. In Baise City, studies specifically documenting edible flowers and their associated traditional knowledge are still scarce. Moreover, quantitative ethnobotanical approaches, including the assessment of cultural importance and use-related indices, have rarely been applied in this area. Consequently, the diversity, traditional uses, and cultural significance of edible flowers among local communities in Baise City remain insufficiently understood.

At the same time, traditional flower-eating practices are undergoing a gradual decline in many regions due to modernization, changing lifestyles, urbanization, and the increasing consumption of processed foods [22]. Nevertheless, these practices continue to persist in certain local communities, where edible flowers remain closely associated with traditional cuisines, ecological knowledge, and cultural heritage [23]. Therefore, documenting and preserving traditional knowledge related to edible flowers is important not only for safeguarding local cultural traditions but also for promoting sustainable utilization of plant resources and supporting the conservation of local food systems [24].

Therefore, the present study aims to investigate edible flowers in Baise City by integrating biodiversity assessment with ethnobotanical analysis. The specific objectives are to: (i) document the diversity of edible flower species, (ii) record traditional knowledge related to their uses, preparation methods, and cultural roles, and (iii) evaluate their ethnobotanical significance using quantitative indices. This study provides new insights into the relationship between plant diversity and traditional knowledge systems and contributes to the conservation and sustainable utilization of edible floral resources.

## 2. Materials and Methods

### 2.1. Study Area

**Figure 1:**
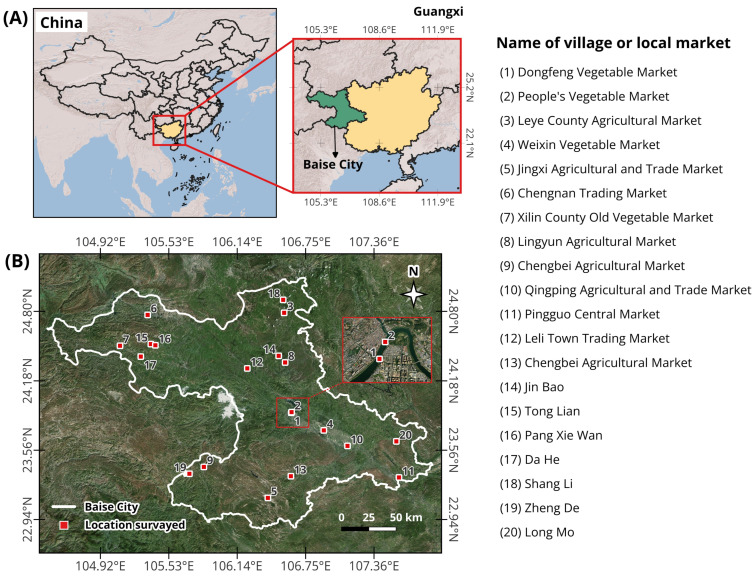
Map of the study area: (**A**) Guangxi Province in China is highlighted in yellow, with the red box indicating the location of Baise City (green); (**B**) surveyed locations in Baise City, where each number corresponds to a village or local market listed alongside the map. (map created with “QGIS” program ver. 3.34 [26], geographic system ID: WGS 84, EPSG 4326).

Baise City is located in the northwestern part of Guangxi Zhuang Autonomous Region, southern China, bordering Vietnam to the southwest and neighboring Yunnan Province to the west (Figure 1). Geographically, the region lies approximately between 22°51′–25°07′ N latitude and 104°28′–107°54′ E longitude. Baise covers a vast area characterized by complex topography, including mountainous terrains, karst landscapes, river valleys, and subtropical forests. The elevation varies considerably across the region, contributing to diverse ecological habitats and rich plant diversity [25].

The climate of Baise City is characterized as subtropical monsoon, with warm temperatures, abundant rainfall, and clearly defined wet and dry seasons. The mean annual temperature ranges from approximately 19–22 °C, while annual precipitation varies between 1100 and 1800 mm. Rainfall is concentrated mainly between April and September, whereas the dry season generally occurs from October to March. These favorable climatic conditions, together with the region’s diverse topography and habitats, support rich plant diversity and provide suitable environments for a wide range of wild and cultivated edible plant species, including edible flowers [27].

Baise City is inhabited by multiple ethnic groups, including the Zhuang, Han, Yao, Miao, and other local communities. These groups possess rich traditional knowledge regarding the utilization of plant resources for food, medicine, and cultural practices. Wild and cultivated edible plants are commonly sold in local markets and are widely incorporated into daily diets and traditional cuisines. Among these resources, edible flowers constitute an important component of local food culture and are used in various forms, such as fresh vegetables, soups, herbal beverages, seasonings, and traditional dishes [28,29].

### 2.2. Plant Collection and Identification

**Figure 2:**
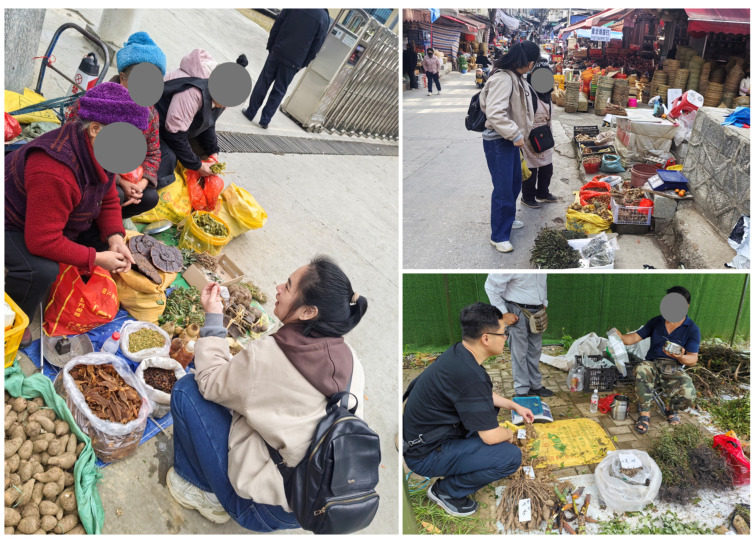
Survey of edible flowers conducted by the authors in local markets of Baise City, Guangxi, China.

Field investigations were conducted in local markets, villages, home gardens, agricultural areas, and surrounding natural habitats in Baise City from March 2025 to February 2026 (Figure 2). Ethnobotanical surveys were carried out through direct observation, semi-structured interviews, and market investigations to document edible flower species and their associated traditional knowledge. Voucher specimens of all recorded species were collected whenever possible during flowering periods to facilitate accurate identification.

For each species, detailed ethnobotanical information was recorded, including local names, growth forms, habitats, edible parts, preparation methods, modes of consumption, medicinal uses, and cultural significance. Fresh specimens were photographed in the field, and representative samples containing flowers and other diagnostic characters were collected and processed according to standard herbarium methods. The prepared voucher specimens were deposited at Baise University, Baise City, Guangxi Zhuang Autonomous Region, China, for proper curation and long-term preservation.

Plant identification was conducted using taxonomic keys, regional floras, herbarium specimens, and relevant botanical literature, including the Flora of China and related references. Scientific names and nomenclature were verified using Plants of the World Online (POWO) [30].

The present study included both wild and cultivated species whose floral parts were consumed as food, beverages, condiments, or supplementary food materials by local communities. Species with additional medicinal functions were also included when their flowers were traditionally consumed. Information related to medicinal applications associated with edible flowers was documented to better understand the relationship between food use and traditional healthcare practices in the study area.

In this study, the term “edible flowers” is used in a broad ethnobotanical sense to include flowers, inflorescences, and other floral structures consumed as food. Inflorescences refer to clustered floral units or composite floral structures that are commonly harvested and consumed as a whole rather than as individual flowers. This definition was applied consistently throughout field documentation, specimen classification, and data analysis.

### 2.3. Ethnobotanical Study

The ethnobotanical component of this study aimed to document traditional knowledge and utilization practices associated with edible flowers in Baise City. Data were collected from February 2025 to January 2026 through semi-structured interviews, informal conversations, participant observation, and market surveys conducted in local villages and traditional markets. A total of 80 informants participated in the study, comprising 40 males and 40 females, aged between 22 and 65 years (Table 1).

Informant selection combined purposive and snowball sampling approaches to ensure the inclusion of individuals possessing direct knowledge of edible flowers and related traditional practices. Priority was given to elders, home cooks, farmers, plant gatherers, herbal practitioners, market vendors, and other community members recognized for their expertise in local food plants. Initial key informants were identified with assistance from village leaders and local residents, while additional participants were recruited through recommendations from previously interviewed informants.

Interviews followed a semi-structured format and focused on vernacular names, edible parts, flowering seasons, habitats, harvesting methods, preparation techniques, modes of consumption, medicinal applications, and cultural significance associated with edible flowers. Information regarding market availability, seasonal trade, preservation practices, and perceived changes in traditional flower consumption was also documented. When possible, demonstrations of flower collection, preparation, and cooking practices were observed directly in households, markets, home gardens, and surrounding natural environments to verify reported uses and improve data reliability.

Participant observation was conducted throughout the fieldwork period to better understand the integration of edible flowers into local food systems and traditional healthcare practices. Repeated market visits were performed to document commercially traded edible flowers, including their forms of sale, frequency of occurrence, and seasonal availability.

Prior to data collection, the objectives and procedures of the study were clearly explained to all participants, and informed consent was obtained in accordance with the ethical guidelines of the International Society of Ethnobiology (ISE) Code of Ethics [31] and the principles of the Nagoya Protocol on Access and Benefit-Sharing [32]. Participation was entirely voluntary, and informants were informed of their right to withdraw from the study at any time without consequence.

Although the present study did not involve sensitive personal data and therefore did not require formal institutional ethical approval, all research activities were conducted in accordance with internationally accepted ethical standards for ethnobotanical research, emphasizing transparency, mutual respect, and reciprocal benefit for local communities.

### 2.4. Data Analysis

Ethnobotanical data obtained from interviews and field observations were organized and analyzed using descriptive statistics and quantitative ethnobotanical indices to evaluate the cultural and medicinal significance of edible flower species in Baise City. Species diversity, growth forms, preparation methods, modes of consumption, and medicinal applications were summarized according to local use patterns. To quantitatively assess the importance of edible flowers within the local communities, the Cultural Food Significance Index (CFSI), Informant Consensus Factor (ICF), and Fidelity Level (FL) were calculated following standard ethnobotanical approaches.

#### 2.4.1. Cultural Food Significance Index (CFSI)

In the present study, the CFSI was employed to evaluate the relative cultural importance of edible flower species used by local communities in Baise City for both dietary and medicinal purposes. The index, originally proposed by Pieroni [33], integrates multiple ethnobotanical indicators into a single quantitative measure reflecting the cultural value of each species. The CFSI was calculated using the following formula:

CFSI = QI × AI × FUI × PUI × MFFI × TSAI × FMRI × 10^−2^ (1)

The Quotation Index (QI) represents the proportion of informants who mentioned a particular species. The Availability Index (AI) reflects the local abundance and accessibility of the species, ranging from very common (4.0) to rare (1.0). The Frequency of Utilization Index (FUI) evaluates how frequently a species is consumed, with values ranging from more than once per week (5.0) to no longer used during the past 30 years (0.5). The Parts Used Index (PUI) measures the diversity of floral and associated plant parts utilized for food purposes. The Multifunctional Food Use Index (MFFI) assesses the diversity of culinary applications, including raw consumption, boiling, frying, steaming, soup preparation, beverages, condiments, and other traditional preparations.

The Taste Score Appreciation Index (TSAI) reflects local perceptions regarding flavor and palatability, with values ranging from terrible (4.0) to excellent (10.0). Finally, the Food-Medicinal Role Index (FMRI) evaluates the degree to which a species is recognized as both a food resource and a medicinal plant, ranging from not recognized (1.0) to very high medicinal importance (5.0). Species with higher CFSI values were considered to possess greater cultural and dietary significance within the study area.

Principal component analysis (PCA) was performed to evaluate the relationships among ethnobotanical indices and plant species. The analysis was conducted using standardized ethnobotanical variables, including FUI, QI, TASI, AI, MFFI, CFSI, FMRI, and PUI. PCA was carried out using OriginPro software version 2018 (OriginLab Corporation, Northampton, MA, USA). The first two principal components (PC1 and PC2) were used to visualize the distribution patterns and correlations among ethnobotanical variables and plant species in a biplot.

#### 2.4.2. Informant Consensus Factor (ICF)

The ICF was employed to assess the degree of agreement among informants regarding the medicinal uses of edible flower species for different ailment categories. This index helps identify categories of diseases for which traditional knowledge and plant use are highly consistent within the community. Fic was calculated following Heinrich et al. [34] using the equation:

(2) $$\mathrm{ICF}\;=\;\frac{n_{\mathrm{ur}}-n_{t}}{n_{\mathrm{ur}}-1}$$

where n_ur_ represents the total number of use reports within a particular medicinal category, and n_t_ denotes the number of species used for that category. ICF values range from 0 to 1, with values approaching 1 indicating a high degree of consensus among informants. High ICF values suggest that certain ailments are well recognized within the community and that specific edible flower species are widely shared and consistently used for their treatment.

#### 2.4.3. Fidelity Level (FL)

The FL was used to determine the relative preference of informants for a particular edible flower species in treating a specific ailment. This index reflects the proportion of informants who consistently cited the same species for the same therapeutic purpose and provides insight into the cultural reliability of traditional medicinal knowledge. FL was calculated following Friedman et al. [35] using the following formula:

(3) $$\mathrm{FL}\;=\;\frac{I_{p}}{I_{u}}\;\times \;100$$

where I_p_ represents the number of informants who cited a species for a specific ailment, and I_u_ is the total number of informants mentioning the species for any medicinal purpose. Species with high FL values were considered to have strong therapeutic specificity and high agreement among informants regarding their medicinal applications.

## 3. Results

### 3.1. Diversity of Edible Flowers

**Figure 3:**
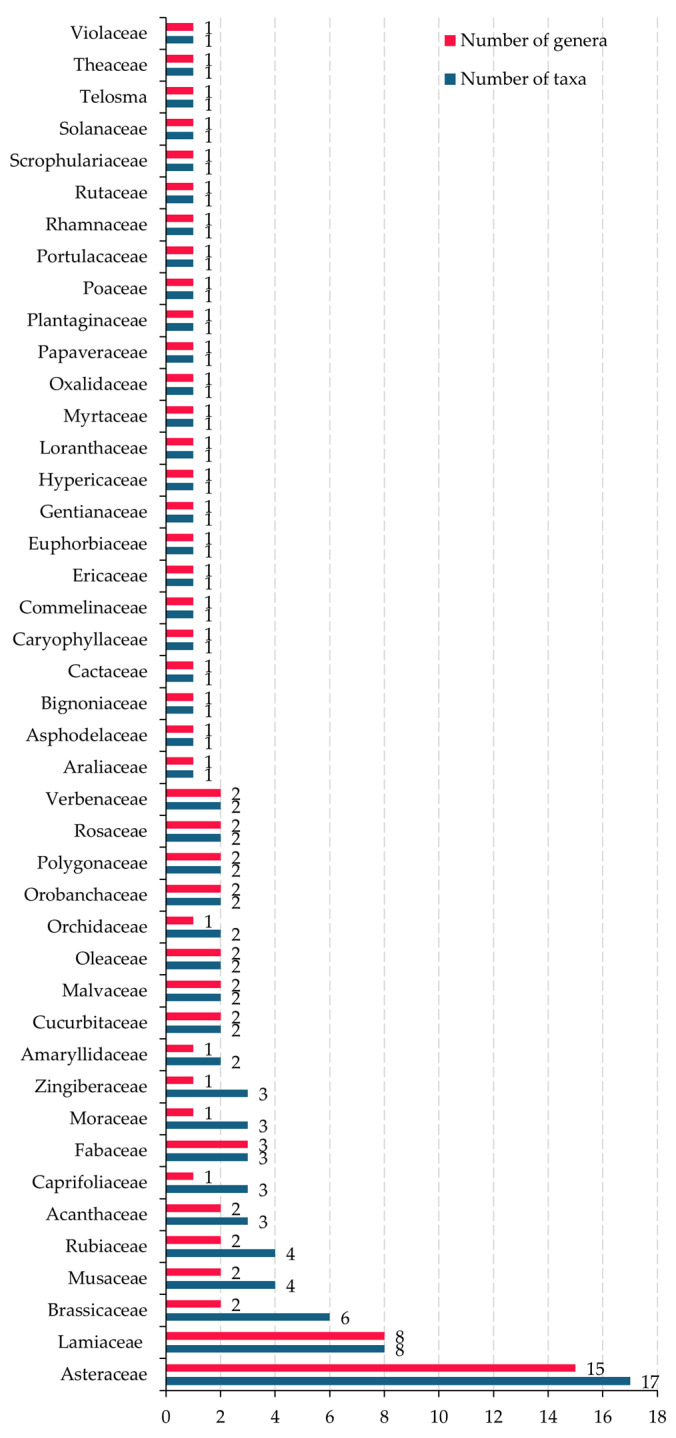
Number of edible flower taxa and genera across plant families documented in Baise City.

A total of 96 edible flower taxa belonging to 77 genera and 44 families were documented in Baise City (Table 2). The taxonomic composition of edible flowers is illustrated in Figure 3, which summarizes the distribution at family and genus levels.

Among the recorded families, Asteraceae was the most represented, followed by Lamiaceae and Brassicaceae. Several other families, including Musaceae, Rubiaceae, Acanthaceae, Caprifoliaceae, Fabaceae, Moraceae, and Zingiberaceae, also contributed multiple taxa, while most remaining families were represented by a single species.

Nine families, including Amaryllidaceae, Cucurbitaceae, Malvaceae, Oleaceae, Orchidaceae, Orobanchaceae, Polygonaceae, Rosaceae, and Verbenaceae, each contained 2 taxa. The remaining families were represented by a single taxon each.

**Figure 4:**
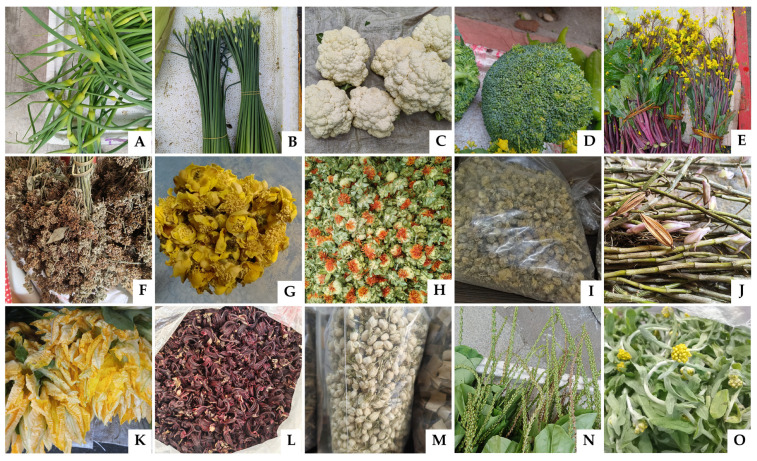
Representative examples of edible flowers in Baise: (**A**) *Allium sativum* L.; (**B**) *Allium tuberosum* Rottler ex Spreng.; (**C**) *Brassica cretica* subsp. *cretica*; (**D**) *Brassica oleracea* L. cv “Italica”; (**E**) *Brassica rapa* L. cv “Purpuraria”; (**F**) *Buddleja officinalis* Maxim.; (**G**) *Camellia pingguoensis* D.Fang; (**H**) *Carthamus tinctorius* L.; (**I**) *Chrysanthemum indicum* L.; (**J**) *Dendrobium aphyllum* (Roxb.) C. E. C. Fisch.; (**K**) *Gynostemma pentaphyllum* (Thunb.) Makino; (**L**) *Hibiscus sabdariffa* L.; (**M**) *Jasminum sambac* (L.) Aiton; (**N**) *Plantago asiatica* L.; (**O**) *Pseudognaphalium affine* (D.Don) Anderb. Photos by Wei Shen.

At the generic level, most genera were represented by a single taxon, reflecting a broad taxonomic distribution of edible flowers recorded in the study area. Overall, the results indicate a high taxonomic dispersion, with most families and genera contributing only a few edible species (Figure 4).

### 3.2. Composition of Edible Flowers

**Figure 5:**
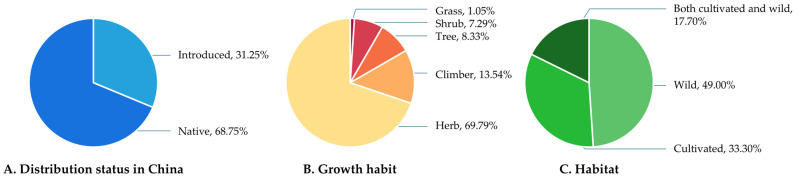
Percentage distribution of edible flowers in Baise, according to (**A**) distribution status in China; (**B**) growth habit; (**C**) habitat.

Among the 96 edible flower taxa documented in Baise City, 66 taxa (68.75%) were native to China, whereas 30 taxa (31.25%) were introduced species (Figure 5). Native species constituted the majority of the recorded edible flowers in the study area.

The documented edible flower species exhibited diverse growth habits (Figure 4). Herbs represented the dominant growth form, comprising 67 taxa (69.79%) of the total recorded species. Climbers were the second most common group with 13 taxa (13.54%), followed by trees with 8 taxa (8.33%) and shrubs with 7 taxa (7.29%). Grass were least represented, accounting for only 1 taxon (1.05%).

Regarding habitat occurrence, 47 taxa (48.96%) were recorded exclusively from wild habitats, representing the largest proportion of edible flower species documented in the study area. Cultivated species accounted for 32 taxa (33.33%), while 17 taxa (17.71%) occurred in both wild and cultivated environments (Figure 4).

### 3.3. Used Parts of Edible Flowers

**Figure 6:**
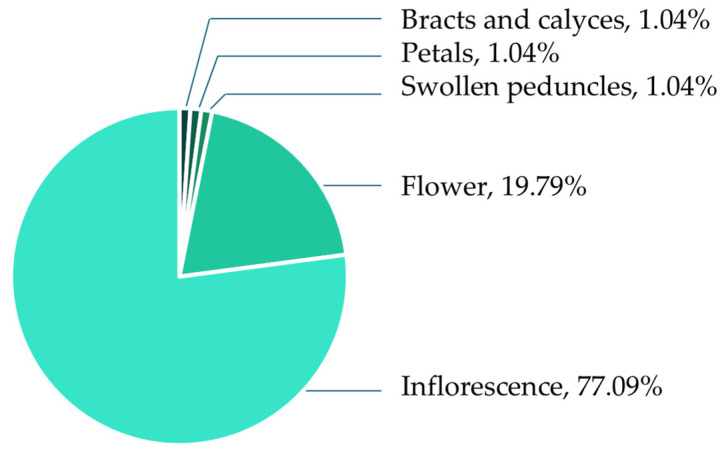
Percentage distribution of utilized parts of edible flowers in Baise City.

Different floral organs were utilized as edible parts among the recorded edible flower species in Baise City (Figure 6). Inflorescences were the most commonly consumed floral organs, accounting for 74 taxa (77.09%) of the total documented species. Whole flowers were utilized in 19 taxa (19.79%). Other floral parts were less frequently consumed, including bracts and calyces, petals, and swollen peduncles, each represented by a single taxon (1.04%).

Many edible flowers recorded in Baise City were consumed together with other plant parts (Table 2). Stems and leaves were the most frequently associated edible organs, followed by young shoots, roots, and leaves. In several species, flowers or inflorescences were also consumed together with bulbs, fleshy taproots, or entire young aerial parts. Nevertheless, some taxa were consumed exclusively as floral organs without accompanying vegetative parts.

### 3.4. Utilization of Edible Flowers

#### 3.4.1. Edible Flowers Used as Condiments and Flavoring

Only one edible flower species documented in Baise City was specifically used as a condiment and flavoring agent (Table 2). The dried flower buds of *Syzygium aromaticum* were commonly added to foods as a seasoning to enhance aroma and flavor. Besides its culinary use, the species was also recognized locally for its medicinal value.

#### 3.4.2. Edible Flowers Used as Food Dyeing

Three edible flower species were recorded as natural food-coloring agents in Baise City (Table 2), namely *B. officinalis*, *C. ternatea*, and *D. tinctoria*. These species were traditionally used to color glutinous rice and various local sweets.

The aerial parts of *D. tinctoria*, including the inflorescences, stems, and leaves, were used as natural coloring materials for glutinous rice preparation. The flowers of *C. ternatea* were widely used to produce a blue color in glutinous rice and traditional desserts. Similarly, the inflorescences of *B. officinalis* were utilized for coloring both glutinous rice and sweet foods. Depending on local practices, these species were used either fresh or dried.

#### 3.4.3. Edible Flowers Used as Herbal Tea

A total of 23 edible flower species were documented as herbal tea plants in Baise City (Table 2). Most were prepared by boiling fresh or dried flowers or inflorescences in water and consumed as beverages. Many of these species were also associated with traditional medicinal uses.

Flowers and inflorescences of species such as *C. pingguoensis*, *C.* × *morifolium*, *J. sambac*, *O. fragrans*, and *R. chinensis* were commonly used for tea preparation. Several other species, including *A. paniculata*, *B. alba*, *B. pilosa*, *C. indicum*, *H. japonicum*, *L. confusa*, *L. hypoglauca*, and *L. macrantha*, were frequently consumed as herbal teas and also recognized in local traditional medicine.

Some species had additional specialized uses. *Panax notoginseng* was valued for its calming properties, whereas *C. tinctorius* was associated with promoting blood circulation. *A. julibrissin* and *C.* × *aurantium* f. *aurantium* were also commonly prepared as herbal teas. Fresh shoots and inflorescences of *D. aphyllum* and *D. devonianum* were boiled together to prepare beverages.

Most herbal tea species were used in dried form, although several taxa, such as *B. alba*, *B. pilosa*, *D. aphyllum*, *D. devonianum*, and *T. mongolicum*, were also consumed fresh. In addition to tea preparation, Hibiscus sabdariffa was also used for food coloring and alcoholic beverage preparation.

#### 3.4.4. Edible Flowers Used as Sweet, Dessert, or Snack

Six edible flower species were recorded as being consumed as sweets, desserts, or snacks (Table 2). Most were eaten fresh and directly consumed without extensive preparation.

The inflorescences of *F. auriculata*, *F. carica*, and *F. tsiangii* were commonly eaten raw, including the fleshy receptacles together with the small internal flowers and seeds. Among these species, *F. carica* was additionally consumed in dried form. Young inflorescences of *I. cylindrica* were also occasionally eaten as snack foods.

The petals of *R. simsii* were consumed fresh and noted for their sour taste. In addition, the swollen peduncles of *H. acerba* were eaten as snacks and also recognized for their traditional medicinal use.

#### 3.4.5. Edible Flowers Used as Vegetables

A total of 31 edible flower species were documented as vegetables in Baise City (Table 2). Most species were consumed after cooking, particularly by stir-frying, boiling, stewing, or cooking in soups and hot pots.

Inflorescences were the most frequently consumed floral organs, although flowers, flower buds, and young aerial parts were also widely utilized. Species such as *A. sativum*, *A. tuberosum*, *Brassica* spp., *Musa* spp., *T. cordata*, and *Zingiber* spp. were commonly prepared as cooked vegetables. In several species, floral organs were consumed together with young shoots, leaves, stems, or whole aerial parts, especially in *C. crepidioides*, *E. sonchifolia*, *G. bicolor*, *P. oleracea*, *S. americanum*, and *S. oleraceus*.

Some species showed distinctive preparation methods. Flowers of *C. moschata* were cooked directly in soups or stuffed with minced meat before boiling. Fresh stamens of *B. ceiba* were stir-fried, blanched, or added to soups, while dried flowers were commonly used in soup preparation. Young aerial parts of *G. pensylvanica* and *P. affine* were pounded and mixed with glutinous rice to prepare traditional green rice cakes.

Most vegetable species were consumed fresh, whereas a few taxa, including *B. ceiba*, *H. citrina*, and *S. undatus*, were utilized in both fresh and dried forms.

#### 3.4.6. Edible Flowers Used as Medicinal Edible Plants

A total of 64 taxa were documented as medicinal edible plants in Baise City (Table 2). Most medicinal edible flowers were consumed in the form of inflorescences, although flowers and other floral structures were also used in several species. In many cases, floral organs were consumed together with stems, leaves, roots, young shoots, or other vegetative parts.

The medicinal edible flower taxa represented a wide range of plant groups. Several species were also recorded in other utilization categories, particularly herbal teas and vegetables, indicating a close relationship between food use and traditional medicinal practices in the study area.

Most medicinal edible flowers were prepared by boiling in water. Some species were additionally used fresh, dried, crushed for external application, stir-fried, or cooked in soups and hot pots. Both fresh and dried materials were commonly utilized depending on the species and preparation method.

Many medicinal edible flowers had multiple functions and overlapped with other categories such as vegetables, herbal teas, condiments, and snacks. This multifunctional use reflects the important role of edible flowers in both daily diets and local traditional knowledge systems in Baise City.

### 3.5. Cultural Significance of Edible Flowers

**Figure 7:**
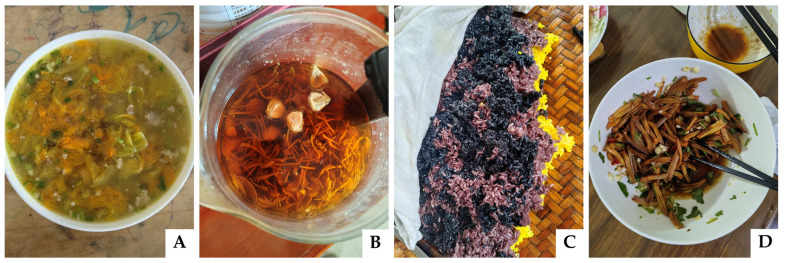
Representative food preparations using edible flowers: (**A**) Soup prepared with *Cucurbita moschata* Duchesne flowers; (**B**) Herbal tea prepared from *Lonicera hypoglauca* Miq.; (**C**) Yellow-colored steamed glutinous rice dyed with *Buddleja officinalis* Maxim.; (**D**) Cold dish prepared with *Bombax ceiba* L. flowers. Photos by Wei Shen.

The cultural food significance index (CFSI) values of edible flowers recorded in Baise City varied considerably among species, reflecting differences in frequency of use, cultural preference, versatility, and perceived importance in local communities (Figure 7 and Table S1). Among all recorded taxa, *P. asiatica* showed the highest CFSI value (684.00), indicating its outstanding cultural importance as an edible flower species in the study area. Other highly ranked species included *E. sonchifolia* (436.80), *S. americanum* (421.20), *S. oleraceus* (312.00), *P. oleracea* (312.00), and *G. bicolor* (288.60). These species were commonly consumed as vegetables and were widely recognized by local informants.

Several medicinal edible flowers also exhibited relatively high CFSI values. *Hypericum japonicum* recorded a CFSI value of 267.90, while *V. inconspicua* reached 207.00 despite having a comparatively lower quotation frequency. Species such as *D. chinensis*, *G. pentaphyllum*, *S. scandens*, and *L. japonicus* also showed notable cultural significance because of their combined food and medicinal roles.

Among vegetable taxa, species of *Brassica* were especially prominent. *B. rapa* cv. “Oleifera”, *B. oleracea* cv. “Albiflora”, and *B. rapa* cv. “Purpuraria” exhibited relatively high CFSI values, reflecting their frequent consumption and broad acceptance as edible flowering vegetables. In addition, *P. affine* and *G. pensylvanica*, which are traditionally used in the preparation of green rice cakes, also ranked highly.

Species used for natural food coloring, herbal teas, and condiments generally showed lower CFSI values than vegetable species, although several taxa remained culturally important. *D. tinctoria*, *B. officinalis*, and *C. ternatea* were valued as natural food-coloring agents, while *O. fragrans*, *J. sambac*, and *R. chinensis* were associated with herbal tea preparation. *Syzygium aromaticum*, the only species recorded as a condiment and flavoring agent, showed moderate cultural significance with a CFSI value of 52.50.

Snack and dessert flowers generally exhibited relatively low CFSI values. Species such as *F. auriculata*, *F. carica*, *F. tsiangii*, *I. cylindrica*, and *R. simsii* were consumed occasionally and had lower quotation frequencies compared with commonly used vegetable taxa.

**Figure 8:**
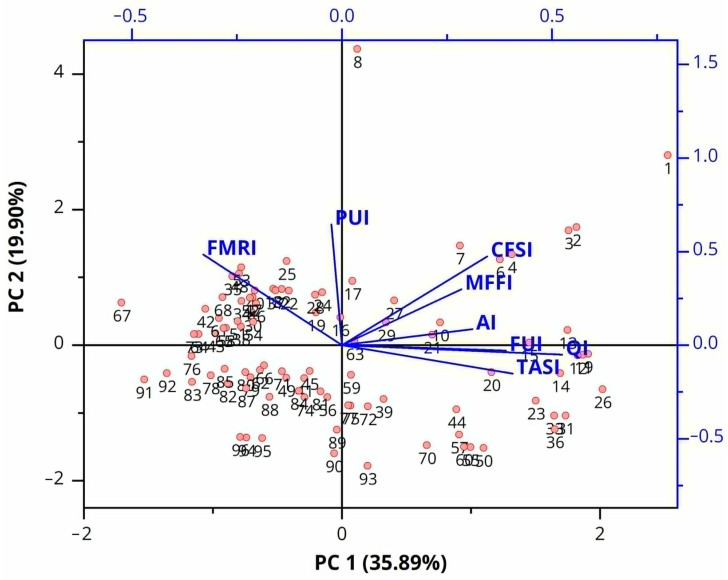
Principal component analysis (PCA) biplot illustrating the relationships among ethnobotanical indices and plant species. Vectors indicate the contribution and direction of ethnobotanical variables, whereas numbered points represent individual plant species (Table S1).

The PCA biplot revealed that PC1 and PC2 explained 35.89% and 19.90% of the total variation, respectively (Figure 8). Variables including FUI, QI, TASI, and AI were positively correlated and strongly associated with samples distributed on the positive side of PC1, indicating plant species with high utilization and cultural importance. In contrast, FMRI showed an opposite trend, suggesting differences between food-medicinal roles and general utilization patterns. The close orientation of MFFI and CFSI vectors indicated a positive relationship between food function and cultural significance.

### 3.6. Ethnomedicinal of Edible Flowers

#### 3.6.1. Fidelity Level of Edible Flowers

The FL analysis showed different degrees of agreement among informants regarding the medicinal uses of edible flowers in Baise City (Table 3). Several species exhibited very high FL values, indicating that informants consistently associated particular taxa with specific therapeutic uses. Species with 100% FL included *A. ciliata* for treating wind-cold syndrome and common cold, *A. pilosa* for physical weakness, *A. decumbens* for stomach disorders, *C. pingguoensis* for high blood pressure, *C. tinctorius* for promoting blood circulation, *H. acerba* and *P. montana* for relieving alcohol intoxication, *M. lasiocarpa* for sore throat, *S. undatus* and *T. mongolicum* for clearing heat, and *Z. mioga* for treating wind-cold conditions.

Many medicinal edible flowers were associated with treatments related to heat-clearing and detoxification. High FL values for these applications were recorded in *E*. *sonchifolia* (83.33%), *M. pubescens* (80.00%), *S. diffusum* (80.00%), *B. pilosa* (75.00%), *C. indicum* (75.00%), *E. hirta* (75.00%), and *L. confusa* (75.00%). These species were widely recognized among local communities and frequently prepared as herbal decoctions or medicinal foods.

Several species showed more than one important medicinal application. *P. capitata*, *P. nodiflora*, *S. plebeia*, *V. officinalis*, and *Y. japonica* were reported for multiple uses, including clearing heat, detoxification, treatment of colds, sore throat, and diuretic purposes. In these taxa, FL values were distributed among several therapeutic categories rather than concentrated in a single use, reflecting their broad role in local traditional medicine.

Species related to blood circulation and women’s health also showed relatively high informant consensus. *C. grandiflora* and *L. japonicus* both had FL values of 60.00% for promoting blood circulation and 40.00% for regulating menstruation. In addition, *E. prostrata* showed notable agreement for blood-related treatments, including cooling the blood (58.33%) and stopping bleeding (41.67%).

Respiratory disorders represented another major therapeutic category. Species such as *A. indica*, *P. frutescens*, *P. asiatica*, *S. cantoniensis*, and *Y. japonica* were commonly used for treating common cold, sore throat, cough, and wind-cold conditions. Among these, *A. indica* showed similar FL values for treating common cold (52.94%) and sore throat (47.06%), indicating that both applications were widely recognized by informants.

#### 3.6.2. Informant Consensus Factor of Edible Flowers

The ICF values indicated a generally high degree of agreement among informants regarding the medicinal uses of edible flowers in Baise City (Table 4). ICF values ranged from 0.839 to 1.000 across the recorded therapeutic categories, suggesting strong consistency in traditional knowledge and medicinal plant selection among local communities.

The highest ICF values (1.000) were recorded for Cardiological Disorders, Eye Disorders, and Urological Disorders. These categories were represented by only one medicinal species in each case, namely *C. pingguoensis* for high blood pressure, *C. × morifolium* for eye health, and *P. capitata* for diuretic purposes. Although the number of taxa was limited, the complete agreement among informants indicates strong recognition of these specific therapeutic uses.

High ICF values were also observed for Blood Disorders (0.942), Obstetrics and Gynecology Disorders (0.933), and Musculoskeletal Disorders (0.923). Species used for promoting blood circulation and regulating menstruation, such as *C. grandiflora*, *C. tinctorius*, and *L. japonicus*, were consistently cited by informants. Similarly, relatively strong agreement was found for taxa used in treating rheumatic pain and relieving pain.

Respiratory Disorders showed a high ICF value of 0.902, based on 113 use reports involving 12 taxa. Species such as *A. ciliata*, *A. indica*, *P. asiatica*, *S. plebeia*, and *Y. japonica* were frequently mentioned for treating common cold, sore throat, cough, and wind-cold conditions. The large number of use reports reflects the importance of respiratory-related treatments in local traditional medicine.

Infection/Immune Disorders had the highest number of use reports (N_ur_ = 394) and taxa (N_t_ = 40), with an ICF value of 0.901. This category included many edible flower species traditionally used for clearing heat, reducing inflammation, and promoting detoxification. Frequently cited taxa included *B. pilosa*, *E. sonchifolia*, *G. bicolor*, *P. oleracea*, and *T. mongolicum*. The high ICF value suggests substantial agreement among informants despite the large number of species involved.

General Tonic also showed a relatively high consensus (ICF = 0.889), including species used for relieving summer heat, dispelling dampness, promoting health, and treating physical weakness. In contrast, Poisoning and Toxicology had the lowest ICF value (0.839), although it still indicated relatively strong agreement considering the high number of taxa (N_t_ = 28) and use reports (N_ur_ = 169). This category mainly included species associated with detoxification and relief of alcohol intoxication.

## 4. Discussion

### 4.1. Diversity and Ethnobotanical Significance of Edible Flowers

Overall, the results indicate that edible flowers in Baise City form an integrated biocultural system in which biodiversity, traditional knowledge, and multifunctional use are closely interconnected rather than being independent components. The present study documented a high diversity of edible flower species in Baise City, Guangxi, China, reflecting the rich biocultural heritage and extensive ethnobotanical knowledge maintained by local communities. The wide range of utilization categories, including vegetables, herbal teas, snacks, condiments, food colorants, and medicinal edible plants, demonstrates the multifunctional role of edible flowers within local food systems. Similar multifunctional uses of edible flowers have been reported in other parts of China [4,21,36] and Southeast Asia [37,38], where edible plants frequently serve overlapping nutritional, medicinal, and cultural functions.

The high representation of families such as Asteraceae, Lamiaceae, Brassicaceae, and Rubiaceae may be associated with their ecological adaptability and abundance in subtropical environments [39]. Many species belonging to these families are widely recognized in traditional medicine and local cuisines because of their aromatic properties, nutritional value, and medicinal potential [40]. In particular, herbaceous species accounted for a large proportion of the recorded taxa, reflecting their accessibility, rapid growth, and continuous availability in agricultural landscapes, roadsides, home gardens, and disturbed habitats [41].

The predominance of native edible flower species is primarily driven by their easy accessibility in natural environments, eliminating the need for market dependence, as well as the long-term transmission of ethnobotanical knowledge through intergenerational cultural learning. In contrast, introduced species have been incorporated into local food systems through processes of globalization and market integration. Rather than replacing traditional resources, these species are selectively adopted, indicating an adaptive and resilient biocultural system in which traditional knowledge and new plant resources coexist dynamically.

The extensive use of edible flowers as vegetables and herbal teas highlights their importance in everyday dietary practices. Several species, including *P. asiatica*, *E. sonchifolia*, *S. americanum*, *S. oleraceus*, and *P. oleracea*, exhibited particularly high Cultural Food Significance Index (CFSI) values, indicating their strong cultural importance and frequent utilization. These species are deeply embedded in local culinary traditions and remain widely available in local markets and household food preparation [42,43].

The use of edible flowers as natural food colorants further illustrates the cultural creativity and ecological knowledge of local communities [44]. Species such as *C. ternatea*, *D. tinctoria*, and *B. officinalis* were traditionally used for coloring glutinous rice and desserts, reflecting the esthetic and symbolic importance of naturally colored foods in local food culture. Such practices also demonstrate the continued relevance of traditional ecological knowledge in daily life [45,46].

### 4.2. Role of Edible Flowers in Food Security and Nutrition

The findings collectively suggest that edible flowers contribute simultaneously to nutritional security, dietary diversification, and resilience of local food systems. Edible flowers documented in this study play an important role in supporting local food and nutritional security. Many recorded species are inexpensive, seasonally abundant, and easily accessible from both cultivated and wild environments. Their continued consumption contributes to dietary diversity and provides supplementary food resources, particularly in rural communities [47].

Several commonly consumed edible flowers are known to contain vitamins, minerals, carbohydrates, anthocyanins, polyphenols, flavonoids, and other bioactive compounds that contribute to human health [48,49]. These compounds are widely recognized for their antioxidant, anti-inflammatory, antimicrobial, and immune-supporting properties, which may help reduce the risk of chronic diseases and support overall well-being. In particular, anthocyanins and polyphenols are associated with strong antioxidant activity and protection against oxidative stress [50], while carbohydrates contribute to energy supply and nutritional value [51]. The presence of these compounds further highlights the importance of edible flowers not only as food resources but also as functional foods with potential therapeutic benefits. Species commonly consumed as vegetables and herbal teas may therefore contribute to improving dietary quality and nutritional diversity within local food systems [52]. Moreover, the frequent use of fresh plant materials reflects the continued reliance on locally available biological resources and demonstrates the importance of traditional plant-based foods in supporting sustainable diets and community health [10].

In the context of increasing environmental uncertainty, climate change, and changes in agricultural production systems, wild and semi-cultivated edible plants may become increasingly important as resilient food resources [53]. Many edible flower species require relatively low management inputs and can grow under diverse environmental conditions. Their integration into local diets therefore contributes to adaptive food systems and may help buffer communities against fluctuations in food availability [54].

The multifunctional nature of edible flowers also strengthens household food security. Several species simultaneously serve as vegetables, herbal teas, medicinal plants, and flavoring agents, allowing communities to maximize the utility of locally available plant resources [55]. This multifunctionality reflects efficient resource use and highlights the close relationship between biodiversity and sustainable livelihoods [56].

### 4.3. Ethnomedicinal Importance and Traditional Healthcare

A clear pattern emerging from the results is the strong food–medicine continuum, where edible flowers function as both dietary components and therapeutic agents within local healthcare systems. The present study demonstrates a strong overlap between food and medicine, a characteristic commonly described as the “food–medicine continuum” in ethnobotanical research [57]. A large proportion of the recorded edible flower species were associated with traditional medicinal applications, particularly for treating infections, respiratory disorders, inflammation, gastrointestinal conditions, and general health maintenance.

The predominance of herbal decoctions and herbal teas as preparation methods reflects the importance of oral administration in traditional healthcare systems [58]. Several species, including *A. paniculata*, *B. alba*, *C. indicum*, *L. confusa*, and *T. mongolicum*, were widely used for clearing heat and detoxification, which are central therapeutic concepts in traditional Chinese medicine [28,59].

The high ICF values observed in several therapeutic categories indicate a strong degree of shared knowledge among informants. Particularly high consensus values for cardiological disorders, eye disorders, blood disorders, and respiratory disorders suggest that local communities possess well-established ethnomedicinal knowledge regarding these health conditions. Similarly, high FL values for several species indicate strong informant agreement concerning specific medicinal applications, which may reflect perceived effectiveness and long-term cultural use [60].

Some medicinal edible flowers also exhibited multifunctional therapeutic roles. Species such as *C. grandiflora*, *C. tinctorius*, and *L. japonicus* were associated with blood circulation and gynecological health, whereas *A. julibrissin* and *P. notoginseng* were linked to calming effects and emotional well-being. These diverse applications illustrate the complexity of local medicinal knowledge systems and the broad healthcare functions of edible flowers in daily life [61,62].

The medicinal significance of edible flowers additionally suggests considerable potential for future phytochemical and pharmacological research. Species exhibiting high FL and ICF values may represent promising candidates for investigations related to antioxidants, antimicrobial, anti-inflammatory, and functional food properties [63,64].

### 4.4. Cultural Transmission and Knowledge Preservation

These results collectively indicate that traditional knowledge remains highly embedded in daily life but is increasingly vulnerable to socio-economic transformation. Traditional knowledge associated with edible flowers in Baise City reflects long-term interactions between local communities and surrounding ecosystems. The persistence of traditional preparation methods, medicinal applications, and food customs demonstrates the continued relevance of ethnobotanical knowledge in local culture [65].

However, traditional knowledge systems are increasingly threatened by modernization, urbanization, changing lifestyles, and dietary transitions [11]. Younger generations may rely more heavily on commercial foods and modern healthcare systems, reducing opportunities for the transmission of traditional ecological knowledge [66]. Migration to urban areas for education and employment may further weaken intergenerational knowledge transfer [67].

At the same time, local markets continue to function as important centers for cultural exchange and knowledge transmission. Many edible flowers recorded in this study were sold in traditional markets, where vendors and consumers exchange information regarding preparation methods, medicinal uses, and seasonal availability. These market systems therefore play an important role in maintaining traditional food knowledge and supporting local plant diversity [68].

The documentation of edible flower knowledge is particularly important because ethnobotanical knowledge is dynamic and context-dependent. Some plant uses may gradually disappear if they are no longer practiced or transmitted within communities. Systematic documentation therefore contributes not only to biodiversity research but also to the preservation of intangible cultural heritage [69].

### 4.5. Conservation, Sustainable Utilization, and Environmental Safety

The results demonstrate that conservation strategies must simultaneously address biodiversity loss, cultural erosion, and environmental safety risks. The high diversity of edible flowers documented in this study highlights the importance of biodiversity conservation in Baise City and surrounding regions. Many edible flower species are collected from wild habitats, forest margins, agricultural areas, and disturbed ecosystems that are increasingly affected by land-use change, habitat degradation, and agricultural intensification [70].

The conservation of edible flowers should therefore integrate both ecological and cultural dimensions. Protecting habitats alone may not be sufficient if traditional knowledge related to plant use continues to decline. Community-based conservation approaches that recognize the cultural importance of edible plants may help strengthen local participation in biodiversity conservation [71].

Species with high cultural importance and frequent utilization may serve as priority taxa for sustainable management and conservation planning. The cultivation of culturally important edible flowers in home gardens or community agricultural systems may reduce harvesting pressure on wild populations while simultaneously supporting household food security and local economies [72].

In addition to ecological sustainability, environmental safety is a critical but often overlooked component of edible flower utilization. Plants collected from roadsides, agricultural fields, and peri-urban or industrial areas may be exposed to heavy metals, pesticides, and other contaminants. These substances can accumulate in plant tissues and potentially enter the human food chain, posing risks to consumer health. Therefore, careful selection of harvesting locations and increased public awareness of environmental quality are essential to ensure the safety of edible flower consumption.

The use of edible flowers as natural food colorants, herbal beverages, and functional foods also presents opportunities for sustainable economic development. Increasing consumer interest in natural products and health-promoting diets may create opportunities for value-added products derived from edible flowers. However, sustainable harvesting practices, habitat protection, and environmental safety considerations should remain central to any future commercialization efforts [73].

### 4.6. Toxicological Safety Considerations of Edible Flowers

The results highlight that edible flower consumption involves both nutritional benefits and species-specific toxicological risks, requiring a balanced safety perspective. Although edible flowers are widely consumed in traditional food systems, several species documented in this study possess inherent toxicological risks that require careful consideration. The safety of consumption depends strongly on species-specific chemical constituents, dosage, and preparation methods.

For example, *S. scandens* contains pyrrolizidine alkaloids known to cause hepatotoxicity, including liver damage, veno-occlusive disease, and potential carcinogenic and teratogenic effects with chronic exposure. *E. hirta* contains diterpene esters and alkaloids that may induce neurological and gastrointestinal disturbances at high doses, despite showing low acute oral toxicity in experimental studies. *S. americanum* contains solanine in unripe berries, which may cause gastrointestinal discomfort, while *C. saxicola* contain isoquinoline alkaloids that may affect the central nervous system at high intake levels. In addition, *H. citrina* requires proper processing, such as blanching or boiling, to reduce potentially harmful compounds prior to consumption.

These findings highlight that traditional edibility does not always equate to complete safety, and appropriate processing methods are essential for reducing toxic risks. Moreover, variation in preparation techniques and consumption habits may significantly influence exposure levels among local communities (Table 5).

### 4.7. Potential for Future Research

The synthesis of findings suggests a need for integrated, multidisciplinary, and applied research frameworks that connect ethnobotany, nutrition, toxicology, and environmental science. The present study provides valuable baseline information for future ethnobotanical, nutritional, phytochemical, and pharmacological investigations. Many edible flower species documented in Baise City remain poorly studied despite their extensive traditional use.

Future studies should evaluate the nutritional composition, bioactive compounds, antioxidant properties, and pharmacological activities of culturally important edible flowers. Comparative studies across different ethnic groups and geographical regions may also improve understanding of knowledge variation and cultural adaptation in edible plant use.

In addition, further research on market systems, cultivation practices, and sustainable harvesting strategies would contribute to the long-term conservation and utilization of edible flower resources. Integrating traditional knowledge with modern scientific approaches may support the development of sustainable food systems, functional foods, and culturally relevant healthcare resources while simultaneously preserving local biocultural diversity.

The advancement and effective implementation of edible flower research would benefit from a multidisciplinary and cross-sectoral approach. This should involve collaboration among ethnobotanists, taxonomists, phytochemists, pharmacologists, toxicologists, nutritionists, and environmental scientists, together with local institutions, universities, and public health authorities. Such integrated cooperation is essential to strengthen conservation planning, food safety assessment, and the development of public awareness programs that promote the sustainable and safe use of edible flowers and traditional food practices.

## 5. Conclusions

This study documented a high diversity of edible floral resources in Baise City, Guangxi, China, comprising 96 taxa belonging to 77 genera and 44 families. Asteraceae, Lamiaceae, and Brassicaceae were the dominant families, while herbaceous species and inflorescences 0 in Baise City function not only as food resources but also as important components of traditional healthcare systems. Several taxa exhibited high CFSI, FL, and ICF values, indicating strong cultural importance, consistent medicinal applications, and substantial informant agreement. Frequently utilized species such as *Plantago asiatica*, *Emilia sonchifolia*, *Solanum americanum*, *Sonchus oleraceus*, and *Portulaca oleracea* remain deeply integrated into local dietary practices and traditional medicine. The overlap between culinary and medicinal applications further supports the concept of a food–medicine continuum within local ethnobotanical knowledge systems.

The predominance of multifunctional species used as vegetables, herbal teas, condiments, medicinal foods, and natural food colorants also highlights the biological and cultural importance of edible floral resources in maintaining dietary diversity and supporting resilient local food systems. In addition, the continued utilization of native wild species reflects the close relationship between biodiversity, ecological knowledge, and traditional subsistence practices among local communities.

However, increasing urbanization, environmental change, and shifts in dietary behavior may gradually reduce the transmission of traditional knowledge associated with edible flowers. Considering the increasing use of wild edible flowers, greater attention should also be given to toxicological safety assessment, potential environmental contamination risks, and public awareness regarding sustainable and safe harvesting practices to ensure their long-term safe integration into local food systems and public health strategies. Furthermore, several documented species possess potential toxicological risks or may accumulate environmental contaminants when harvested from polluted habitats, highlighting the need for future safety evaluations and conservation-oriented management approaches.

Overall, this study provides important baseline biological and ethnobotanical data for future nutritional, phytochemical, pharmacological, ecological, and conservation-related investigations of edible flowers in southern China. The preservation and sustainable utilization of edible floral resources may contribute to biodiversity conservation, food security, and the long-term maintenance of regional biocultural diversity.

## Figures and Tables

**Table 1:** Demographic and cultural characteristics of surveyed villages and local markets in Baise City, including GPS coordinates, gender, ethnicity, and languages of informants.

Name of Village or Local Market	GPS Coordinates		Gender		Ethnicity	Language
	Longitude (E, W)	Latitude (N, S)	Male	Female		
Chengbei Agricultural Market	105.84253192°	23.41046936°	2	2	Han, Zhuang	Mandarin, Zhuang
Chengbei Agricultural Market	106.61841452°	23.32753557°	2	2	Han, Zhuang	Mandarin, Zhuang
Chengnan Trading Market	105.34140408°	24.76826286°	2	2	-	Mandarin
Da He	105.28010488°	24.39716477°	2	2	Zhuang	Mandarin, Zhuang
Dongfeng Vegetable Market	106.62231982°	23.89454374°	2	2	-	Mandarin
Jin Bao	106.50972068°	24.40389667°	2	2	Yao	Mandarin, Yao
Jingxi Agricultural and Trade Market	106.4134267°	23.13306168°	2	2	-	Mandarin
Leli Town Trading Market	106.22978389°	24.2915986°	2	2	Han, Yao, Zhuang	Mandarin, Yao, Zhuang
Leye County Agricultural Market	106.55837461°	24.78707059°	2	2	-	Mandarin
Lingyun Agricultural Market	106.5673399°	24.34466516°	2	2	Han, Yao, Zhuang	Mandarin, Yao, Zhuang
Long Mo	107.55889893°	23.63888271°	2	2	Han	Mandarin
Pang Xie Wan	105.41010618°	24.4968339°	2	2	Han	Mandarin
People’s Vegetable Market	106.62441194°	23.90075533°	2	2	-	Mandarin
Pingguo Central Market	107.58225024°	23.31630648°	2	2	-	Mandarin
Qingping Agricultural and Trade Market	107.12322772°	23.59835071°	2	2	-	Mandarin
Shang Li	106.54974461°	24.90300026°	2	2	Zhuang	Mandarin, Zhuang
Tong Lian	105.36965579°	24.50757282°	2	2	Miao	Mandarin, Miao
Weixin Vegetable Market	106.9120574°	23.73731089°	2	2	Han, Zhuang	Mandarin, Zhuang
Xilin County Old Vegetable Market	105.09474277°	24.49243556°	2	2	-	Mandarin
Zheng De	105.71358472°	23.34925459°	2	2	Zhuang	Mandarin, Zhuang

**Table 2:** Documented edible flower species in Baise City, Guangxi, China.

Family	Scientific Name	Chinese Name	Vernacular Name	DiC	GH	Habitat	Edible Parts of the Floral Organ	With Other Parts When Consuming Flowers	Utilization	CFSI	Voucher Number
Acanthaceae	*Andrographis paniculata* (Burm.f.) Wall. ex Nees	穿心莲 (chuān xīn lián)	jīn dāo wā (Y), chān xīn liān (Z), chuān xīn lián(D)	IT	Hb	Bt	Inflorescence	Always shoot and leaf	HT, MP	29.25	XC-WS 0191
Acanthaceae	*Dicliptera chinensis* (L.) Juss.	狗肝菜 (gǒu gān cài)	bū yāng wā (Y), gūo xiē zhēng (Z), gǒu gān cài (D)	NT	Hb	Bt	Inflorescence	Always young shoot	MP, VG	117.60	XC-WS 0255
Acanthaceae	*Dicliptera tinctoria* (Nees) Kostel.	观音草 (guān yīn cǎo)	wā gān (Y), gūo zhēng (Z), lán zǐ cǎo(D)	NT	Hb	Ct	Inflorescence	Always shoot and leaf	FD	87.36	XC-WS 0202
Amaryllidaceae	*Allium sativum* L.	蒜 (suàn)	diè yī (M), guō suī (Z), suàn tái (D)	IT	Hb	Ct	Inflorescence	-	VG	8.82	XC-WS 0259
Amaryllidaceae	*Allium tuberosum* Rottler ex Spreng.	韭 (jiǔ)	Lao (M), biē jiē (Z), jiǔ cài tái (D)	IT	Hb	Ct	Inflorescence	-	VG	4.14	XC-WS 0168
Araliaceae	*Panax notoginseng* (Burkill) F.H.Chen	三七 (sān qī)	tíng qī (M), wā dián qī (Z), tián qī(D)	NT	Hb	Ct	Inflorescence	-	HT, MP	1.80	XC-WS 0186
Asphodelaceae	*Hemerocallis citrina* Baroni	黄花菜 (huáng huā cài)	wā jīn jīn (Z), huáng huā cài (D)	NT	Hb	Ct	Flower	-	VG	12.42	XC-WS 0189
Asteraceae	*Acmella ciliata* (Kunth) Cass.	天文草 (tiān wén cǎo)	jīn niǔ kòu (D)	IT	Hb	Wd	Inflorescence	Always shoot and leaf	MP	7.31	XC-WS 0239
Asteraceae	*Bidens alba* (L.) DC.	大白花鬼针草 (dà bái huā guǐ zhēn cǎo)	wā zà (Y), ān chāng (M), fēn liē ān (Z), guǐ zhēn cǎo (D)	IT	Hb	Wd	Inflorescence	Always root, shoot, and leaf	HT, MP	34.20	XC-WS 0256
Asteraceae	*Bidens pilosa* L.	鬼针草 (guǐ zhēn cǎo)	wā zà (Y), ān chāng (M), fēn liē ān (Z), guǐ zhēn cǎo (D)	IT	Hb	Wd	Inflorescence	Always root, shoot, and leaf	HT, MP	7.13	XC-WS 0213
Asteraceae	*Blumea megacephala* (Randeria) C.T.Chang & C.H.Yu	东风草 (dōng fēng cǎo)	dōng fēng cǎo (D)	NT	Hb	Wd	Inflorescence	Always shoot and leaf	MP	25.59	XC-WS 0203
Asteraceae	*Carthamus tinctorius* L.	红花 (hóng huā)	hóng huā (D)	IT	Hb	Ct	Inflorescence	-	HT, MP	2.25	XC-WS 0251
Asteraceae	*Chrysanthemum* × *morifolium* (Ramat.) Hemsl.	菊花 (jú huā)	yīng wèi (Y), wā zhōu (Z), gòng jú (D)	NT	Hb	Ct	Inflorescence	-	HT, MP	2.48	XC-WS 0249
Asteraceae	*Chrysanthemum indicum* L.	野菊 (yě jú)	yē yīng wèi (Y), yē wā zhōu (Z), yě jú huā (D)	NT	Hb	Wd	Inflorescence	-	HT, MP	2.93	XC-WS 0181
Asteraceae	*Crassocephalum crepidioides* (Benth.) S.Moore	野茼蒿 (yě tóng hāo)	yōng wā (Y), yōu gāi hōu (M), biē gā long (Z), hóng jūn cài (D)	IT	Hb	Wd	Inflorescence	Always young shoot	VG	46.20	XC-WS 0250
Asteraceae	*Eclipta prostrata* (L.) L.	鳢肠 (lǐ cháng)	guō biē mēng (Z), hàn lián cǎo (D)	IT	Hb	Wd	Inflorescence	Always shoot and leaf	MP	3.90	XC-WS 0240
Asteraceae	*Emilia sonchifolia* (L.) DC.	一点红 (yī diǎn hóng)	a dī mū suì (Y), biē ruì líng (Z), yī diǎn hóng (D)	NT	Hb	Bt	Inflorescence	Always young shoot and leaf	MP, VG	436.80	XC-WS 0248
Asteraceae	*Gamochaeta pensylvanica* (Willd.) Cabrera	匙叶合冠鼠曲 (chí yè hé guān shǔ qū)	Wèn (Y), yōu nīng (M), guō bù yìn hān (Z), shǔ qú cǎo (D)	IT	Hb	Wd	Inflorescence	Always young shoot and leaf	VG	79.56	XC-WS 0257
Asteraceae	*Gynura bicolor* (Roxb. ex Willd.) DC.	红凤菜 (hóng fèng cài)	wā gē (Y), biē dūn jīng (Z), zǐ bèi cài (D)	NT	Hb	Bt	Inflorescence	Always young leaf and shoot	MP, VG	288.60	XC-WS 0217
Asteraceae	*Pseudognaphalium affine* (D.Don) Anderb.	鼠曲草 (shǔ qū cǎo)	Wèn (Y), yōu nīng (M), yìn hān (Z), shǔ qú cǎo (D)	NT	Hb	Wd	Inflorescence	Always young shoot and leaf	VG	109.20	XC-WS 0170
Asteraceae	*Senecio scandens* Buch.-Ham. ex D.Don	千里光 (qiān lǐ guāng)	xīan lǐ guāng (Z), qiān lǐ guāng (D)	NT	Hb	Wd	Inflorescence	Always shoot and leaf	MP	73.13	XC-WS 0219
Asteraceae	*Sonchus oleraceus* L.	苦苣菜 (kǔ jù cài)	wā yīn (Y), yōu āi (M), kǔ jù cài (D)	IT	Hb	Bt	Inflorescence	Always young leaf and shoot	MP, VG	312.00	XC-WS 0183
Asteraceae	*Taraxacum mongolicum* Hand.-Mazz.	蒲公英 (pú gōng yīng)	yē yě zāi (Y), yōu āi (M), pú gōng yīng (D)	NT	Hb	Bt	Inflorescence	Always root and leaf	HT, MP	57.38	XC-WS 0201
Asteraceae	*Youngia japonica* (L.) DC.	黄鹌菜 (huáng ān cài)	yē yě (Y), yōu āi (M), bié wā hěn (Z), yě cài (D)	NT	Hb	Wd	Inflorescence	Always root, shoot and leaf	MP	45.60	XC-WS 0265
Bignoniaceae	*Campsis grandiflora* (Thunb.) K.Schum.	凌霄 (líng xiāo)	líng xiāo huā (D)	NT	Cl	Ct	Flower	-	MP	0.90	XC-WS 0231
Brassicaceae	*Brassica cretica* subsp. *cretica*	花椰菜 (huā yé cài)	yóu kū (M), huā yé cài (D)	IT	Hb	Ct	Inflorescence	-	VG	24.96	XC-WS 0226
Brassicaceae	*Brassica oleracea* L. cv “Albiflora”	白花甘蓝 (bái huā gān lán)	yóu gā (M), gāi lán wā (Z), gān lán cài (D)	IT	Hb	Ct	Inflorescence	Always young shoot and leaf	VG	116.48	XC-WS 0208
Brassicaceae	*Brassica oleracea* L. cv “Italica”	西蓝花 (xī lán huā)	yóu kū (M), gāi lán wā (Z), xī lán huā (D)	IT	Hb	Ct	Inflorescence	-	VG	27.36	XC-WS 0241
Brassicaceae	*Brassica rapa* L. cv “Oleifera”	芸薹 (yún tái)	yóu gā (M), biē dāi (Z), cài xīn (D)	IT	Hb	Ct	Inflorescence	Always young shoot and leaf	VG	120.64	XC-WS 0216
Brassicaceae	*Brassica rapa* L. cv “Purpuraria”	紫菜薹 (zǐ cài tái)	yóu gā (M), gāi lán wā (Z), zǐ cài tái (D)	IT	Hb	Ct	Inflorescence	Always young shoot and leaf	VG	112.32	XC-WS 0224
Brassicaceae	*Capsella bursa-pastoris* (L.) Medik.	荠 (jì)	biě děng dāng (Z), jì cài (D)	NT	Hb	Wd	Inflorescence	Always root and leaf	VG	7.20	XC-WS 0176
Cactaceae	*Selenicereus undatus* (Haw.) D.R.Hunt	量天尺 (liàng tiān chǐ)	hūo lōng guò (M), wā lōng gúo (Z), bà wáng huā (D)	IT	Cl	Ct	Flower	-	MP, VG	12.15	XC-WS 0233
Caprifoliaceae	*Lonicera confusa* (Sweet) DC.	华南忍冬 (huá nán rěn dōng)	jīn yīn huā (Y), jīn yīng huà (M), jiā shā long (Z)	NT	Cl	Bt	Flower	-	HT, MP	9.45	XC-WS 0218
Caprifoliaceae	*Lonicera hypoglauca* Miq.	菰腺忍冬 (gū xiàn rěn dōng)	jīn yīn huā (Y), jīn yīng huà (M), jiā shā long (Z)	NT	Cl	Bt	Flower	-	HT, MP	13.05	XC-WS 0243
Caprifoliaceae	*Lonicera macrantha* (D.Don) Spreng.	大花忍冬 (dà huā rěn dōng)	jīn yīn huā (Y), jīn yīng huà (M), jiā shā long (Z)	NT	Cl	Bt	Flower	-	HT, MP	1.80	XC-WS 0187
Caryophyllaceae	*Stellaria media* (L.) Vill.	繁缕 (fán lǚ)	yē cán (Y), yóu bīng (M), guō biē yīn (Z), é cháng cài (D)	NT	Hb	Wd	Inflorescence	Always young shoot and leaf	VG	53.04	XC-WS 0225
Commelinaceae	*Commelina communis* L.	鸭跖草 (yā zhí cǎo)	yóu mū lāi āo (M), biē zhā (Z), yā zhí cǎo (D)	NT	Hb	Wd	Inflorescence	Always young shoot and leaf	MP	11.70	XC-WS 0190
Cucurbitaceae	*Cucurbita moschata* Duchesne	南瓜 (nán guā)	ā dōng (M), wā guā (Z), nán guā (D)	IT	Cl	Ct	Flower	-	VG	41.76	XC-WS 0227
Cucurbitaceae	*Gynostemma pentaphyllum* (Thunb.) Makino	绞股蓝 (jiǎo gǔ lán)	yē yā (Y), máng biá (M), guō long (Z), jiǎo gǔ lán (D)	NT	Cl	Bt	Inflorescence	Always young shoot and leaf	HT, MP	76.05	XC-WS 0223
Ericaceae	*Rhododendron simsii* Planch.	杜鹃 (dù juān)	gē shān fang (Y), bāng yē āi (M), yìng shān hóng (D)	NT	Sb	Wd	Petals	-	SW	0.08	XC-WS 0232
Euphorbiaceae	*Euphorbia hirta* L.	飞扬草 (fēi yáng cǎo)	guō háo luō ruǐ (Z), fēi yáng cǎo (D)	IT	Hb	Wd	Inflorescence	Always root, shoot and leaf	MP	12.83	XC-WS 0175
Fabaceae	*Albizia julibrissin* Durazz.	合欢 (hé huan)	hé huan huā (D)	NT	Tr	Ct	Flower	-	HT, MP	1.35	XC-WS 0193
Fabaceae	*Clitoria ternatea* L.	蝶豆 (dié dòu)	dié dòu huā (Z), dié dòu huā (D)	NT	Cl	Ct	Flower	-	FD	7.43	XC-WS 0228
Fabaceae	*Pueraria montana* (Lour.) Merr.	山葛 (shān gé)	Māng (M), yě gě (D)	NT	Cl	Wd	Flower	-	HT, MP	4.05	XC-WS 0238
Gentianaceae	*Metagentiana rhodantha* (Franch.) T.N.Ho & S.W.Liu	红花龙胆 (hóng huā lóng dǎn)	lóng dǎn cǎo (D)	NT	Hb	Wd	Inflorescence	Always root, shoot and leaf	MP	10.69	XC-WS 0171
Hypericaceae	*Hypericum japonicum* Thunb.	地耳草 (dì ěr cǎo)	guō shén lǔ (Z), tián jī huáng (D)	NT	Hb	Wd	Inflorescence	Always root, shoot and leaf	HT, MP	267.90	XC-WS 0194
Lamiaceae	*Agastache rugosa* (Fisch. & C.A.Mey.) Kuntze	藿香 (huò xiāng)	biē guō huō (Z), huò xiāng (D)	IT	Hb	Ct	Inflorescence	Always shoot and leaf	MP	5.85	XC-WS 0180
Lamiaceae	*Anisomeles indica* (L.) Kuntze	广防风 (guǎng fáng fēng)	xiē jī mā (Z), guǎng fáng fēng (D)	NT	Hb	Wd	Inflorescence	Always shoot and leaf	MP	18.28	XC-WS 0177
Lamiaceae	*Elsholtzia cyprianii* (Pavol.) C.Y.Wu & S.Chow	野草香 (yě cǎo xiāng)	yě bó hé (D)	NT	Hb	Wd	Inflorescence	Always shoot and leaf	MP	5.85	XC-WS 0247
Lamiaceae	*Leonurus japonicus* Houtt.	益母草 (yì mǔ cǎo)	yì mǔ cǎo (D)	NT	Hb	Wd	Inflorescence	Always shoot and leaf	MP	51.19	XC-WS 0254
Lamiaceae	*Perilla frutescens* (L.) Britton	紫苏 (zǐ sū)	ā biā (M), biē suī sǔo (Z), zǐ sū (D)	NT	Hb	Ct	Inflorescence	Always shoot and leaf	MP	8.78	XC-WS 0221
Lamiaceae	*Prunella vulgaris* L.	夏枯草 (xià kū cǎo)	xià kū cǎo (D)	NT	Hb	Wd	Inflorescence		MP	6.75	XC-WS 0244
Lamiaceae	*Salvia plebeia* R. Br.	荔枝草 (lì zhī cǎo)	xiē jī mā (Z), lì zhī cǎo (D)	NT	Hb	Wd	Inflorescence	Always shoot and leaf	MP	12.68	XC-WS 0266
Lamiaceae	*Ajuga decumbens* Thunb.	金疮小草 (jīn chuāng xiǎo cǎo)	kū cāo (M), sàn xuè cǎo (D)	NT	Hb	Wd	Inflorescence	Always shoot and leaf	MP	21.94	XC-WS 0195
Loranthaceae	*Macrosolen cochinchinensis* (Lour.) Tiegh.	鞘花 (qiào huā)	jī sheng (M), guō xiàng (Z), jì sheng (D)	NT	Sb	Wd	Inflorescence	Always shoot and leaf	MP	7.80	XC-WS 0263
Malvaceae	*Bombax ceiba* L.	木棉 (mù mián)	mù mián (M), guō gūi wā (Z), mù mián (D)	NT	Tr	Bt	Flower	-	MP, VG	6.00	XC-WS 0253
Malvaceae	*Hibiscus sabdariffa* L.	玫瑰茄 (méi guī qié)	luò shén huā (D)	IT	Hb	Ct	Bracts and calyces	-	HT	31.19	XC-WS 0236
Moraceae	*Ficus auriculata* Lour.	大果榕 (dà guǒ róng)	yē biáo (Y), jī ān dōng (M), mā wō (Z), dà guǒ róng (D)	NT	Tr	Wd	Inflorescence	-	SW	0.14	XC-WS 0246
Moraceae	*Ficus carica* L.	无花果 (wú huā guǒ)	yē biáo (Y), jī ān dōng (M), mā móu (Z), wú huā guǒ (D)	IT	Sb	Ct	Inflorescence	-	SW	0.54	XC-WS 0178
Moraceae	*Ficus tsiangii* Merr. ex Corner	岩木瓜 (yán mù guā)	yē biáo (Y), jī ān dōng (M), yán mù guā (D)	NT	Tr	Wd	Inflorescence	-	SW	0.10	XC-WS 0220
Musaceae	*Musa* × *paradisiaca* L.	大蕉 (dà jiāo)	jī zēi (M), bǐ zúi (Z), bā jiao (D)	IT	Hb	Ct	Inflorescence	-	VG	2.84	XC-WS 0192
Musaceae	*Musa acuminata* Colla	小果野蕉 (xiǎo guǒ yě jiāo)	jī zēi (M), bǐ zúi (Z), bā jiao (D)	NT	Hb	Wd	Inflorescence	-	VG	2.97	XC-WS 0173
Musaceae	*Musa balbisiana* Colla	野蕉 (yě jiāo)	jī zēi (M), bǐ zúi (Z), bā jiao (D)	NT	Hb	Wd	Inflorescence	-	VG	4.05	XC-WS 0196
Musaceae	*Musella lasiocarpa* (Franch.) C.Y.Wu ex H.W.Li	地涌金莲 (dì yǒng jīn lián)	dì yǒng jīn lián (D)	NT	Hb	Ct	Inflorescence	-	MP	0.34	XC-WS 0169
Myrtaceae	*Syzygium aromaticum* (L.) Merr. & L.M.Perry	丁香蒲桃 (dīng xiāng pú táo)	dīng xiāng (M), dīng xiāng (D),	IT	Tr	Ct	Flower	-	CF, MP	52.50	XC-WS 0197
Oleaceae	*Jasminum sambac* (L.) Aiton	茉莉花 (mò lì huā)	mò lì huā (M), wā mò lì (Z), mò lì huā (D)	IT	Sb	Ct	Flower	-	HT	3.60	XC-WS 0211
Oleaceae	*Osmanthus fragrans* Lour.	木樨 (mù xī)	guō lín gǎi (Z), guì huā (D)	NT	Tr	Ct	Flower	-	HT	15.12	XC-WS 0182
Orchidaceae	*Dendrobium aphyllum* (Roxb.) C. E. C. Fisch.	兜唇石斛 (dōu chún shí hú)	shí hú (Z), shí hú (D)	NT	Hb	Wd	Inflorescence	Alway shoot	HT, MP	5.25	XC-WS 0179
Orchidaceae	*Dendrobium devonianum* Paxton	齿瓣石斛 (chǐ bàn shí hú)	shí hú (Z), shí hú (D)	NT	Hb	Ct	Inflorescence	Alway shoot	HT, MP	2.10	XC-WS 0210
Orobanchaceae	*Aeginetia indica* L.	野菰 (yě gū)	ā biā (M), yān dǒu huā (D)	NT	Hb	Wd	Flower	-	MP	0.34	XC-WS 0252
Orobanchaceae	*Striga asiatica* (L.) Kuntze	独脚金 (dú jiǎo jīn)	gān jī cǎo (D)	NT	Hb	Wd	Inflorescence	Always root, shoot and leaf	MP	59.85	XC-WS 0200
Oxalidaceae	*Oxalis debilis* Kunth	红花酢浆草 (hóng huā cù jiāng cǎo)	wā biē (Y), sūan long (Z), sūan mī mī (D)	IT	Hb	Wd	Inflorescence	Always fleshy taproot, bulb, and leaf	MP, VG	36.00	XC-WS 0229
Papaveraceae	*Corydalis saxicola* Bunting	石生黄堇 (shí shēng huáng jǐn)	kǔ huáng lián (D)	NT	Hb	Bt	Inflorescence	Always root, shoot and leaf	MP	4.28	XC-WS 0258
Plantaginaceae	*Plantago asiatica* L.	车前草 (chē qián cǎo)	mā dēi wā (Y), yōu mū bā (M), biē bō mà (Z), chē qián cǎo (D)	NT	Hb	Bt	Inflorescence	Always root and leaf	MP, VG	684.00	XC-WS 0237
Poaceae	*Imperata cylindrica* (L.) Raeusch.	白茅 (bái máo)	gán dōng (Y), ā biā (M), guō lā kà (Z), bái máo cǎo (D)	NT	Gr	Wd	Inflorescence	-	SW	0.17	XC-WS 0234
Polygalaceae	*Salomonia cantoniensis* Lour.	齿果草 (chǐ guǒ cǎo)	xiāng gēn cǎo (D)	NT	Hb	Wd	Inflorescence	Always root, shoot and leaf	MP	8.02	XC-WS 0235
Polygonaceae	*Persicaria capitata* (Buch.-Ham. ex D. Don) H. Gross	头花蓼 (tóu huā liǎo)	guō fěi (Z), tóu huā liǎo (D)	NT	Hb	Wd	Inflorescence	Always shoot and leaf	MP	14.63	XC-WS 0242
Portulacaceae	*Portulaca oleracea* L.	马齿苋 (mǎ chǐ xiàn)	mā biē yē (Y), ā biā (M), bēi gǔō gi (Z), mǎ chǐ xiàn (D)	IT	Hb	Wd	Inflorescence	Always young shoot and leaf	MP, VG	312.00	XC-WS 0166
Rhamnaceae	*Hovenia acerba* Lindl.	枳椇 (zhǐ jǔ)	zēi yā biáo (Y), guǎi zǎo (M), mā shā jìn (Z), guǎi zǎo (D)	NT	Tr	Bt	Swollen peduncles	-	MP, SW	7.65	XC-WS 0214
Rosaceae	*Agrimonia pilosa* Ledeb.	龙牙草 (lóng yá cǎo)	guō dōng á (Z), xiān hè cǎo (D)	NT	Hb	Wd	Inflorescence	Always root, shoot and leaf	MP	42.75	XC-WS 0215
Rosaceae	*Rosa chinensis* Jacq.	月季花 (yuè jì huā)	yē jī bang (M), wā yuè jì (Z), yuè jì huā (D)	IT	Sb	Ct	Flower	-	HT	1.92	XC-WS 0260
Rubiaceae	*Mussaenda divaricata* Hutch.	展枝玉叶金花 (zhǎn zhī yù yè jīn huā)	wēi yōng fang (Y), yù yè jīn huā (D)	NT	Cl	Wd	Inflorescence	Always root, shoot and leaf	MP	4.28	XC-WS 0222
Rubiaceae	*Mussaenda kwangsiensis* H.L.Li	广西玉叶金花 (guǎng xī yù yè jīn huā)	wēi yōng fang (Y), yù yè jīn huā (D)	NT	Cl	Wd	Inflorescence	Always root, shoot and leaf	MP	4.28	XC-WS 0172
Rubiaceae	*Mussaenda pubescens* W.T.Aiton	玉叶金花 (yù yè jīn huā)	wēi yōng fang (Y), yù yè jīn huā (D)	NT	Cl	Wd	Inflorescence	Always root, shoot and leaf	MP	11.40	XC-WS 0184
Rubiaceae	*Scleromitrion diffusum* (Willd.) R. J. Wang	白花蛇舌草 (bái huā shé shé cǎo)	guō līng lē (Z), bái huā shé shé cǎo (D)	NT	Hb	Wd	Inflorescence	Always shoot and leaf	MP	16.09	XC-WS 0199
Rutaceae	*Citrus* × *aurantium* f. *aurantium*	代代酸橙 (dài dài suān chéng)	wā mā gān (Z), suān chéng huā (D)	IT	Tr	Ct	Flower	-	HT, MP	1.80	XC-WS 0165
Scrophulariaceae	*Buddleja officinalis* Maxim.	密蒙花 (mì méng huā)	lēng wāng fān (Y), gbāng cōu (M), gō wā yǎ (Z), mì méng huā (D)	NT	Sb	Bt	Inflorescence	-	FD	21.60	XC-WS 0230
Solanaceae	*Solanum americanum* Mill.	少花龙葵 (shǎo huā lóng kuí)	biē huā cài (M), biě dāi ēn (Z), bái huā cài (D)	NT	Hb	Bt	Inflorescence	Always young shoot and leaf	MP, VG	421.20	XC-WS 0185
Telosma	*Telosma cordata* (Burm.f.) Merr.	夜来香 (yè lái xiāng)	yè lái xiāng (D)	NT	Cl	Ct	Inflorescence	-	VG	7.20	XC-WS 0174
Theaceae	*Camellia pingguoensis* D.Fang	平果金花茶 (píng guǒ jīn huā chá)	jīn huā chá (D)	NT	Sb	Ct	Flower	-	HT, MP	0.90	XC-WS 0212
Verbenaceae	*Phyla nodiflora* (L.) Greene	过江藤 (guò jiāng téng)	guò jiāng téng (D)	NT	Hb	Wd	Inflorescence	Always root, shoot and leaf	MP	8.55	XC-WS 0264
Verbenaceae	*Verbena officinalis* L.	马鞭草 (mǎ biān cǎo)	má píng mī (Y), mǎ biān cǎo (D)	NT	Hb	Wd	Inflorescence	Always root, shoot and leaf	MP	23.51	XC-WS 0267
Violaceae	*Viola inconspicua* Blume	长萼堇菜 (cháng è jǐn cài)	dē dīng wā (Y), bēi bā séi (Z), dì ding (D)	NT	Hb	Wd	Flower	Always root, shoot and leaf	MP, VG	207.00	XC-WS 0198
Zingiberaceae	*Zingiber mioga* (Thunb.) Roscoe	蘘荷 (ráng hé)	wēng song (Y), kāi niū (M), náng hé (D)	NT	Hb	Wd	Inflorescence	-	MP	1.35	XC-WS 0204
Zingiberaceae	*Zingiber officinale* Roscoe	姜 (jiāng)	Song (Y), kāi (M), híng (Z)	IT	Hb	Ct	Inflorescence	-	VG	0.81	XC-WS 0188
Zingiberaceae	*Zingiber striolatum* Diels	阳荷 (yáng hé)	yng hùo yē (Y), yáng huò (M), làng (Z), yáng hé (D)	NT	Hb	Bt	Inflorescence	-	VG	7.74	XC-WS 0167

Abbreviation. Vernacular name: D (Mandarin) M (Miao language), Y (Yao language), Z (Zuang language). Distribution status in China (DiC): IT (introduced), NT (native). Growth habit (GH): Cl (climber), Gr (grass), Hb (herb), Sb (shrub), Tr (tree). Habitat: Bt (both cultivated and wild), Ct (cultivated), Wd (wild). Utilization: CF (condiments and flavoring), FD (food dyeing), HT (herbal tea), MP (medicinal edible plants), SW (sweets/desserts/snacks), VG (vegetable). CFSI (Cultural Food Significance Index).

**Table 3:** Ethnomedicinal uses of the recorded edible flower species in Baise City, Guangxi, China.

Scientific Name	FL	Used Part	CoP	Preparation	Traditional Application	Therapeutic Categories
*Acmella ciliata* (Kunth) Cass.	100.00	If	D, F	B	T34	Respiratory Disorders
*Aeginetia indica* L.	52.94	Fw	F	B	T1	Respiratory Disorders
	47.06	Fw	F	B	T30	Respiratory Disorders
*Agastache rugosa* (Fisch. & C.A.Mey.) Kuntze	58.33	If, Le, St	D	B	T22	General Tonic
	41.67	If, Le, St	D	B	T21	General Tonic
*Agrimonia pilosa* Ledeb.	100.00	If, Le St	D	B	T28	General Tonic
*Ajuga decumbens* Thunb.	100.00	If, Le St	D, F	B	T31	Gastrointestinal Disorders
*Albizia julibrissin* Durazz.	75.00	Fw	D	B	T5	Neurological Disorders
	25.00	Fw	D	B	T18	Neurological Disorders
*Andrographis paniculata* (Burm.f.) Wall. ex Nees	66.67	If, Le, St	D	B	T6	Infection/Immune Disorders
	33.33	If, Le, St	D	B	T8	Poisoning and Toxicology
*Anisomeles indica* (L.) Kuntze	56.25	If, Le, St	D, F	B	T6	Infection/Immune Disorders
	43.75	If, Le, St	D, F	B	T8	Poisoning and Toxicology
*Bidens alba* (L.) DC.	68.75	If, Le, Rt, St	D, F	B	T6	Infection/Immune Disorders
	31.25	If, Le, Rt, St	D, F	B	T8	Poisoning and Toxicology
*Bidens pilosa* L.	75.00	If, Le, Rt, St	D, F	B	T6	Infection/Immune Disorders
	25.00	If, Le, Rt, St	D, F	B	T8	Poisoning and Toxicology
*Blumea megacephala* (Randeria) C.T.Chang & C.H.Yu	55.56	If, Le, St	D, F	B	T6	Infection/Immune Disorders
	44.44	If, Le, St	D, F	B	T8	Poisoning and Toxicology
*Bombax ceiba* L.	65.00	Fw	D, F	P	T6	Infection/Immune Disorders
	35.00	Fw	D, F	P	T22	General Tonic
*Camellia pingguoensis* D.Fang	100.00	Fw	D	B	T2	Cardiological Disorders
*Campsis grandiflora* (Thunb.) K.Schum.	60.00	Fw	D	B	T11	Blood Disorders
	40.00	Fw	D	B	T14	Obstetrics and Gynecology Disorders
*Carthamus tinctorius* L.	100.00	If	D	B	T11	Blood Disorders
*Chrysanthemum* × *morifolium* (Ramat.) Hemsl.	78.57	If	D	B	T6	Infection/Immune Disorders
	21.43	If	D	B	T9	Eye Disorders
*Chrysanthemum indicum* L.	75.00	If	D	B	T6	Infection/Immune Disorders
	25.00	If	D	B	T8	Poisoning and Toxicology
*Citrus* × *aurantium* f. *aurantium*	70.00	Fw	D	B	T15	General Tonic
	30.00	Fw	D	B	T18	Neurological Disorders
*Commelina communis* L.	66.67	If, Le, St	D, F	B	T6	Infection/Immune Disorders
	33.33	If, Le, St	D, F	B	T8	Poisoning and Toxicology
*Corydalis saxicola* Bunting	78.57	If, Le, Rt, St	D	B	T6	Infection/Immune Disorders
	21.43	If, Le, Rt, St	D	B	T8	Poisoning and Toxicology
*Dendrobium aphyllum* (Roxb.) C. E. C. Fisch.	53.85	If, St	F	B	T10	Gastrointestinal Disorders
	46.15	If, St	F	B	T19	General Tonic
*Dendrobium devonianum* Paxton	57.14	If, St	F	B	T10	Gastrointestinal Disorders
	42.86	If, St	F	B	T19	General Tonic
*Dicliptera chinensis* (L.) Juss.	60.00	If, St	F	P	T6	Infection/Immune Disorders
	40.00	If, St	F	P	T8	Poisoning and Toxicology
*Eclipta prostrata* (L.) L.	58.33	If, Le, St	D, F	B	T7	Blood Disorders
	41.67	If, Le, St	D, F	B	T23	Blood Disorders
*Elsholtzia cyprianii* (Pavol.) C.Y.Wu & S.Chow	75.00	If, Le, St	D, F	B	T21	General Tonic
	25.00	If, Le, St	D, F	B	T22	General Tonic
*Emilia sonchifolia* (L.) DC.	83.33	If, Le, St	F	P	T6	Infection/Immune Disorders
	16.67	If, Le, St	F	P	T3	Infection/Immune Disorders
*Euphorbia hirta* L.	75.00	If, Le, Rt, St	D, F	B	T6	Infection/Immune Disorders
	25.00	If, Le, Rt, St	D, F	B	T17	Respiratory Disorders
*Gynostemma pentaphyllum* (Thunb.) Makino	68.75	If, Le, St	D	B	T6	Infection/Immune Disorders
	31.25	If, Le, St	D	B	T12	General Tonic
*Gynura bicolor* (Roxb. ex Willd.) DC.	66.67	If, Le, St	F	P	T6	Infection/Immune Disorders
	33.33	If, Le, St	F	P	T3	Infection/Immune Disorders
*Hovenia acerba* Lindl.	100.00	Sp	F	E	T16	Poisoning and Toxicology
*Hypericum japonicum* Thunb.	53.85	If, Le, St	D	B	T6	Infection/Immune Disorders
	46.15	If, Le, St	D	B	T8	Poisoning and Toxicology
*Leonurus japonicus* Houtt.	60.00	If, Le, St	D, F	B	T11	Blood Disorders
	40.00	If, Le, St	D, F	B	T14	Obstetrics and Gynecology Disorders
*Lonicera confusa* (Sweet) DC.	75.00	Fw	D	B	T6	Infection/Immune Disorders
	25.00	Fw	D	B	T8	Poisoning and Toxicology
*Lonicera hypoglauca* Miq.	53.33	Fw	D	B	T6	Infection/Immune Disorders
	46.67	Fw	D	B	T8	Poisoning and Toxicology
*Lonicera macrantha* (D.Don) Spreng.	70.00	Fw	D	B	T6	Infection/Immune Disorders
	30.00	Fw	D	B	T8	Poisoning and Toxicology
*Macrosolen cochinchinensis* (Lour.) Tiegh.	65.00	If, Le, St	F	B	T35	Respiratory Disorders
	35.00	If, Le, St	F	B	T29	Musculoskeletal Disorders
*Metagentiana rhodantha* (Franch.) T.N.Ho & S.W.Liu	54.55	If, Le, Rt, St	D, F	B	T6	Infection/Immune Disorders
	45.45	If, Le, Rt, St	D, F	B	T8	Poisoning and Toxicology
*Musella lasiocarpa* (Franch.) C.Y.Wu ex H.W.Li	100.00	If	F	B	T30	Respiratory Disorders
*Mussaenda divaricata* Hutch.	53.85	If, Le, Rt, St	D	B	T6	Infection/Immune Disorders
	46.15	If, Le, Rt, St	D	B	T8	Poisoning and Toxicology
*Mussaenda kwangsiensis* H.L.Li	57.14	If, Le, Rt, St	D	B	T6	Infection/Immune Disorders
	42.86	If, Le, Rt, St	D	B	T8	Poisoning and Toxicology
*Mussaenda pubescens* W.T.Aiton	80.00	If, Le, Rt, St	D	B	T6	Infection/Immune Disorders
	20.00	If, Le, Rt, St	D	B	T8	Poisoning and Toxicology
*Oxalis debilis* Kunth	57.14	Bb, If, Le, Tr	F	P	T6	Infection/Immune Disorders
	42.86	Bb, If, Le, Tr	F	P	T3	Infection/Immune Disorders
*Panax notoginseng* (Burkill) F.H.Chen	58.33	If	D	B	T6	Infection/Immune Disorders
	41.67	If	D	B	T4	Neurological Disorders
*Perilla frutescens* (L.) Britton	54.55	If, Le, St	D, F	B	T25	Respiratory Disorders
	45.45	If, Le, St	D, F	B	T27	Infection/Immune Disorders
*Persicaria capitata* (Buch.-Ham. ex D. Don) H. Gross	40.91	If, Le, St	D, F	B	T6	Infection/Immune Disorders
	31.82	If, Le, St	D, F	B	T8	Poisoning and Toxicology
	27.27	If, Le, St	D, F	B	T26	Urological Disorders
*Phyla nodiflora* (L.) Greene	39.13	If, Le, Rt, St	F	B	T6	Infection/Immune Disorders
	30.43	If, Le, Rt, St	F	B	T8	Poisoning and Toxicology
	21.74	If, Le, Rt, St	F	B	T25	Respiratory Disorders
	8.70	If, Le, Rt, St	F	B	T30	Respiratory Disorders
*Plantago asiatica* L.	71.43	If, Le, Rt	F	P	T25	Respiratory Disorders
	28.57	If, Le, Rt	F	P	T30	Respiratory Disorders
*Portulaca oleracea* L.	75.00	If, Le, St	F	P	T6	Infection/Immune Disorders
	25.00	If, Le, St	F	P	T8	Poisoning and Toxicology
*Prunella vulgaris* L.	58.33	If	D, F	B	T6	Infection/Immune Disorders
	41.67	If	D, F	B	T13	Infection/Immune Disorders
*Pueraria montana* (Lour.) Merr.	100.00	Fw	D	B	T16	Poisoning and Toxicology
*Salomonia cantoniensis* Lour.	76.47	If, Le, Rt, St	D, F	B	T30	Respiratory Disorders
	23.53	If, Le, Rt, St	D, F	B	T32	Gastrointestinal Disorders
*Salvia plebeia* R. Br.	38.46	If, Le, St	F	B	T6	Infection/Immune Disorders
	34.62	If, Le, St	F	B	T8	Poisoning and Toxicology
	19.23	If, Le, St	F	B	T25	Respiratory Disorders
	7.69	If, Le, St	F	B	T30	Respiratory Disorders
*Scleromitrion diffusum* (Willd.) R. J. Wang	80.00	If, Le, St	D, F	B	T6	Infection/Immune Disorders
	20.00	If, Le, St	D, F	B	T8	Poisoning and Toxicology
*Selenicereus undatus* (Haw.) D.R.Hunt	100.00	Fw	D, F	P	T6	Infection/Immune Disorders
*Senecio scandens* Buch.-Ham. ex D.Don	75.00	If, Le, St	D, F	B	T6	Infection/Immune Disorders
	25.00	If, Le, St	D, F	B	T8	Poisoning and Toxicology
*Solanum americanum* Mill.	50.00	If, Le, St	F	P	T6	Infection/Immune Disorders
	50.00	If, Le, St	F	P	T3	Infection/Immune Disorders
*Sonchus oleraceus* L.	60.00	If, Le, St	F	P	T6	Infection/Immune Disorders
	40.00	If, Le, St	F	P	T3	Infection/Immune Disorders
*Striga asiatica* (L.) Kuntze	40.91	If, Le, Rt, St	D, F	B	T27	Infection/Immune Disorders
	31.82	If, Le, Rt, St	D, F	B	T3	Infection/Immune Disorders
	27.27	If, Le, Rt, St	D, F	B	T24	General Tonic
*Syzygium aromaticum* (L.) Merr. & L.M.Perry	56.25	Fw	D	S	T36	Gastrointestinal Disorders
	43.75	Fw	D	S	T20	Musculoskeletal Disorders
*Taraxacum mongolicum* Hand.-Mazz.	100.00	If, Le, Rt	D, F	B	T6	Infection/Immune Disorders
*Verbena officinalis* L.	34.62	If, Le, Rt, St	F	B	T6	Infection/Immune Disorders
	26.92	If, Le, Rt, St	F	B	T8	Poisoning and Toxicology
	23.08	If, Le, Rt, St	F	B	T6	Infection/Immune Disorders
	15.38	If, Le, Rt, St	F	B	T8	Poisoning and Toxicology
*Viola inconspicua* Blume	58.33	Fw, Le, Rt	F	P	T6	Infection/Immune Disorders
	41.67	Fw, Le, Rt	F	P	T8	Poisoning and Toxicology
*Youngia japonica* (L.) DC.	38.10	If, Le, St	F	B	T6	Infection/Immune Disorders
	28.57	If, Le, St	F	B	T8	Poisoning and Toxicology
	23.81	If, Le, St	F	B	T25	Respiratory Disorders
	9.52	If, Le, St	F	B	T30	Respiratory Disorders
*Zingiber mioga* (Thunb.) Roscoe	100.00	If	F	B	T33	Respiratory Disorders

Abbreviation. FL (fidelity level); Used part: Bb (bulb), Fw (flower), If (inflorescence), Le (leaf), Rt, (root), Sp (swollen peduncle), St (shoot), Tr (taproot). Condition of plant material (CoP): D (dry), F (fresh). Preparation: B (Boiling in water and drinking as an herbal decoction), E (eaten raw), P (used as food preparation), S (used as a culinary seasoning in traditional food preparation). T1–T36 indicate standardized traditional medicinal application codes described in the supplementary glossary (Table S2).

**Table 4:** Informant consensus factor (ICF) values for therapeutic categories of medicinal edible flowers recorded in Baise City, Guangxi, China.

Therapeutic Categories	N_ur_	N_t_	ICF
Cardiological Disorders	15	1	1.000
Eye Disorders	3	1	1.000
Urological Disorders	6	1	1.000
Blood Disorders	53	4	0.942
Obstetrics and Gynecology Disorders	16	2	0.933
Musculoskeletal Disorders	14	2	0.923
Neurological Disorders	24	3	0.913
Gastrointestinal Disorders	43	5	0.905
Respiratory Disorders	113	12	0.902
Infection/Immune Disorders	394	40	0.901
General Tonic	73	9	0.889
Poisoning and Toxicology	169	28	0.839

**Table 5:** Summarizes the toxic or potentially adverse effects of selected edible flower species and their associated health risks.

Scientific Name	Toxic Parts/Compounds	Effects/Precautions
*Corydalis saxicola* Bunting	Whole plant; isoquinoline alkaloids	Generally low toxicity. Toxicological studies show toxicity is mild and reversible in animals. However, high doses may cause adverse effects on the central nervous system and organs due to the alkaloid content [74].
*Euphorbia hirta* L.	Whole plant; diterpene esters (phytochemicals), tannins, alkaloids	LD50 > 5000 mg/kg in rats, indicating low acute oral toxicity. However, high doses can cause CNS depression (analgesia, loss of muscle coordination), cerebral hemorrhage, and acute hemorrhagic enteritis (bleeding in intestines) [75].
*Hemerocallis citrina* Baroni	Uncooked flowers; colchicine	Flowers must be blanched (briefly boiled) before consumption to remove toxins. Generally recognized as safe when cooked [76].
*Senecio scandens* Buch.-Ham. ex D.Don	Whole plant (especially leaves); Pyrrolizidine alkaloids (PAs): Senecionine, Seneciphylline, Jacozine	Hepatotoxic (liver-toxic). PAs cause liver damage (veno-occlusive disease), are potentially carcinogenic and teratogenic. Chronic exposure leads to cirrhosis [77]. Do not take large doses when taking it [78].
*Solanum americanum* Mill.	Unripe (green) berries; solanine	The green berries contain solanine, which can cause gastrointestinal discomfort (nausea, vomiting, abdominal pain) when consumed in excess. The toxicity of mature berries is reduced, but they should still be consumed with caution [79].

## Data Availability

The data presented in this study are available on request from the first, last, and corresponding author.

## Supplementary Materials

The following supporting information can be downloaded at: https://www.mdpi.com/article/10.3390/biology15110873/s1, Table S1: Cultural Food Significance Index (CFSI) evaluation of edible flowers in Baise City, Guangxi, China; Table S2: Standardized traditional medicinal application codes (T1–T36) used for ethnomedicinal categorization of edible flower species recorded in Baise City, Guangxi, China.
