# Supplementary material for: Traditional Knowledge and Biocultural Roles of Edible Flowers in Local Food Systems of Baise City, Guangxi, China
Source: Biology (Basel). 2026 Jun 1;15(11):873. doi: 10.3390/biology15110873 (PMC13255686; doi:10.3390/biology15110873)
Supplement: Supplementary file 1 [file biology-15-00873-s001.zip › biology-4361319-supplementary.pdf]

biology

MDPI

Supplementary

Traditional Knowledge and Biocultural Roles of Edible Flowers in Local Food Systems of Baise City, Guangxi, China

Table S1. Cultural Food Significance Index (CFSI) evaluation of edible flowers in Baise City, Guangxi, China

No.	Scientific name	QI	AI	FUI	PUI	MFFI	TASI	FMRI	CFSI
1	*Plantago asiatica* L.	57	4	2	3.75	2	4	5	684.00
2	*Emilia sonchifolia* (L.) DC.	56	3	2	3.25	2	4	5	436.80
3	*Solanum americanum* Mill.	54	3	2	3.25	2	4	5	421.20
4	*Sonchus oleraceus* L.	40	3	2	3.25	2	4	5	312.00
5	*Portulaca oleracea* L.	40	3	2	3.25	2	4	5	312.00
6	*Gynura bicolor* (Roxb. ex Willd.) DC.	37	3	2	3.25	2	4	5	288.60
7	*Hypericum japonicum* Thunb.	47	4	2	4.75	1	3	5	267.90
8	*Viola inconspicua* Blume	10	3	1	17.25	2	4	5	207.00
9	*Brassica rapa* L. cv "Oleifera"	58	4	2	3.25	2	4	1	120.64
10	*Dicliptera chinensis* (L.) Juss.	28	3	2	1.75	2	4	5	117.60
11	*Brassica oleracea* L. cv "Albiflora"	56	4	2	3.25	2	4	1	116.48
12	*Brassica rapa* L. cv "Purpuraria"	54	4	2	3.25	2	4	1	112.32
13	*Pseudognaphalium affine* (D.Don) Anderb.	35	4	2	3.25	3	4	1	109.20
14	*Dicliptera tinctoria* (Nees) Kostel.	56	4	2	3.25	1.5	4	1	87.36
15	*Gamochaeta pensylvanica* (Willd.) Cabrera	34	3	2	3.25	3	4	1	79.56
16	*Gynostemma pentaphyllum* (Thunb.) Makino	26	3	2	3.25	1	3	5	76.05
17	*Senecio scandens* Buch.-Ham. ex D.Don	10	3	2	3.25	2.5	3	5	73.13
18	*Striga asiatica* (L.) Kuntze	28	2	1	4.75	1.5	3	5	59.85
19	*Taraxacum mongolicum* Hand.-Mazz.	17	3	2	3.75	1	3	5	57.38
20	*Stellaria media* (L.) Vill.	34	3	2	3.25	2	4	1	53.04
21	*Syzygium aromaticum* (L.) Merr. & L.M.Perry	35	4	1	0.75	2.5	4	5	52.50
22	*Leonurus japonicus* Houtt.	21	2	1	3.25	2.5	3	5	51.19
23	*Crassocephalum crepidioides* (Benth.) S.Moore	55	3	2	1.75	2	4	1	46.20
24	*Youngia japonica* (L.) DC.	8	4	2	4.75	1	3	5	45.60
25	*Agrimonia pilosa* Ledeb.	8	3	1	4.75	2.5	3	5	42.75
26	*Cucurbita moschata* Duchesne	58	4	2	0.75	3	4	1	41.76
27	*Oxalis debilis* Kunth	3	4	2	3.75	2	4	5	36.00
28	*Bidens alba* (L.) DC.	6	4	2	4.75	1	3	5	34.20
29	*Hibiscus sabdariffa* L.	33	3	1	0.75	3.5	3	4	31.19
30	*Andrographis paniculata* (Burm.f.) Wall. ex Nees	15	3	2	3.25	1	2	5	29.25
31	*Brassica oleracea* L. cv "Italica"	57	4	2	0.75	2	4	1	27.36

32	*Blumea megacephala* (Randeria) C.T.Chang & C.H.Yu	7	3	1	3.25	2.5	3	5	25.59
33	*Brassica cretica* subsp. *cretica*	52	4	2	0.75	2	4	1	24.96
34	*Verbena officinalis* L.	11	3	1	4.75	1	3	5	23.51
35	*Ajuga decumbens* Thunb.	9	3	1	3.25	2.5	2	5	21.94
36	*Buddleja officinalis* Maxim.	60	4	2	0.75	1.5	4	1	21.60
37	*Anisomeles indica* (L.) Kuntze	5	3	1	3.25	2.5	3	5	18.28
38	*Scleromitrion diffusum* (Willd.) R. J. Wang	11	3	1	3.25	1	3	5	16.09
39	*Osmanthus fragrans* Lour.	21	3	2	0.75	1	4	4	15.12
40	*Persicaria capitata* (Buch.-Ham. ex D. Don) H. Gross	8	3	0.5	3.25	2.5	3	5	14.63
41	*Lonicera hypoglauca* Miq.	29	2	2	0.75	1	3	5	13.05
42	*Euphorbia hirta* L.	9	2	1	4.75	1	3	5	12.83
43	*Salvia plebeia* R. Br.	13	2	1	3.25	1	3	5	12.68
44	*Hemerocallis citrina* Baroni	46	3	2	0.75	2	3	1	12.42
45	*Selenicereus undatus* (Haw.) D.R.Hunt	18	3	2	0.75	1	3	5	12.15
46	*Commelina communis* L.	4	3	1	3.25	2	3	5	11.70
47	*Mussaenda pubescens* W.T.Aiton	4	4	1	4.75	1	3	5	11.40
48	*Metagentiana rhodantha* (Franch.) T.N.Ho & S.W.Liu	3	2	1	4.75	2.5	3	5	10.69
49	*Lonicera confusa* (Sweet) DC.	21	2	2	0.75	1	3	5	9.45
50	*Allium sativum* L.	49	3	2	0.75	1	4	1	8.82
51	*Perilla frutescens* (L.) Britton	9	4	0.5	3.25	1	3	5	8.78
52	*Phyla nodiflora* (L.) Greene	3	4	1	4.75	1	3	5	8.55
53	*Salomonia cantoniensis* Lour.	3	3	0.5	4.75	2.5	3	5	8.02
54	*Macrosolen cochinchinensis* (Lour.) Tiegh.	4	4	1	3.25	1	3	5	7.80
55	*Zingiber striolatum* Diels	43	3	2	0.75	1	4	1	7.74
56	*Hovenia acerba* Lindl.	34	3	1	0.75	0.5	4	5	7.65
57	*Clitoria ternatea* L.	55	3	1	0.75	1.5	4	1	7.43
58	*Acmella ciliata* (Kunth) Cass.	5	3	1	3.25	1	3	5	7.31
59	*Capsella bursa-pastoris* (L.) Medik.	12	2	1	3.75	2	4	1	7.20
60	*Telosma cordata* (Burm.f.) Merr.	40	3	2	0.75	1	4	1	7.20
61	*Bidens pilosa* L.	5	1	2	4.75	1	3	5	7.13
62	*Prunella vulgaris* L.	20	3	1	0.75	1	3	5	6.75
63	*Bombax ceiba* L.	4	4	1	0.75	2.5	4	5	6.00
64	*Agastache rugosa* (Fisch. & C.A.Mey.) Kuntze	6	2	1	3.25	1	3	5	5.85
65	*Elsholtzia cyprianii* (Pavol.) C.Y.Wu & S.Chow	4	3	1	3.25	1	3	5	5.85
66	*Dendrobium aphyllum* (Roxb.) C. E. C. Fisch.	10	3	0.5	1.75	1	4	5	5.25
67	*Corydalis saxicola* Bunting	9	1	1	4.75	1	2	5	4.28
68	*Mussaenda divaricata* Hutch.	3	4	0.5	4.75	1	3	5	4.28
69	*Mussaenda kwangsiensis* H.L.Li	3	4	0.5	4.75	1	3	5	4.28

https://doi.org/10.3390/biology15110873

70	*Allium tuberosum* Rottler ex Spreng.	23	3	2	0.75	1	4	1	4.14
71	*Pueraria montana* (Lour.) Merr.	6	3	2	0.75	1	3	5	4.05
72	*Musa balbisiana* Colla	30	3	1	0.75	2	3	1	4.05
73	*Eclipta prostrata* (L.) L.	4	2	1	3.25	1	3	5	3.90
74	*Jasminum sambac* (L.) Aiton	10	3	1	0.75	1	4	4	3.60
75	*Musa acuminata* Colla	22	3	1	0.75	2	3	1	2.97
76	*Chrysanthemum indicum* L.	13	3	1	0.75	1	2	5	2.93
77	*Musa × paradisiaca* L.	21	3	1	0.75	2	3	1	2.84
78	*Chrysanthemum × morifolium* (Ramat.) Hemsl.	11	2	1	0.75	1	3	5	2.48
79	*Carthamus tinctorius* L.	5	2	2	0.75	1	3	5	2.25
80	*Dendrobium devonianum* Paxton	3	2	1	1.75	1	4	5	2.10
81	*Rosa chinensis* Jacq.	4	4	1	0.75	1	4	4	1.92
82	*Panax notoginseng* (Burkill) F.H.Chen	8	1	2	0.75	1	3	5	1.80
83	*Lonicera macrantha* (D.Don) Spreng.	16	1	1	0.75	1	3	5	1.80
84	*Citrus × aurantium* f. *aurantium*	3	2	2	0.75	1	4	5	1.80
85	*Albizia julibrissin* Durazz.	4	3	1	0.75	1	3	5	1.35
86	*Zingiber mioga* (Thunb.) Roscoe	4	3	1	0.75	1	3	5	1.35
87	*Campsis grandiflora* (Thunb.) K.Schum.	3	2	1	0.75	1	4	5	0.90
88	*Camellia pingguoensis* D.Fang	3	1	2	0.75	1	4	5	0.90
89	*Zingiber officinale* Roscoe	6	3	2	0.75	1	3	1	0.81
90	*Ficus carica* L.	12	3	1	0.75	0.5	4	1	0.54
91	*Musella lasiocarpa* (Franch.) C.Y.Wu ex H.W.Li	6	1	0.5	0.75	1	3	5	0.34
92	*Aeginetia indica* L.	3	2	0.5	0.75	1	3	5	0.34
93	*Imperata cylindrica* (L.) Raeusch.	3	3	1	0.75	0.5	5	1	0.17
94	*Ficus auriculata* Lour.	8	3	0.5	0.75	0.5	3	1	0.14
95	*Ficus tsiangii* Merr. ex Corner	3	3	1	0.75	0.5	3	1	0.10
96	*Rhododendron simsii* Planch.	5	3	0.5	0.75	0.5	3	1	0.08

Abbreviation. QI (Quotation Index); AI (Availability Index); FUI (Frequency of Utilization Index); PUI (Parts Used Index); MFFI (Multifunctional Food Use Index); TSAI (Taste Score Appreciation Index); FMRI (Food-Medicinal Role Index); CFSI (Cultural Food Significance Index).

Table S2. Standardized traditional medicinal application codes (T1–T36) used for ethnomedicinal categorization of edible flower species recorded in Baise City, Guangxi, China.

Code	Standardized Traditional Application
T1	Used in traditional medicine for treating wind-cold syndrome and common cold.
T2	Used in traditional medicine as a health-promoting tea for high blood pressure.
T3	Used in traditional medicine for treating inflammation.
T4	Used in traditional medicine for calming effects.
T5	Used in traditional medicine for calming the mind.
T6	Used in traditional medicine for clearing heat.
T7	Used in traditional medicine for cooling the blood.
T8	Used in traditional medicine for detoxification.
T9	Used in traditional medicine for improving eye health.
T10	Used in traditional medicine for nourishing the stomach.

https://doi.org/10.3390/biology15110873

T11	Used in traditional medicine for promoting blood circulation.
T12	Used in traditional medicine for promoting general health.
T13	Used in traditional medicine for reducing swelling.
T14	Used in traditional medicine for regulating menstruation.
T15	Used in traditional medicine for regulating qi.
T16	Used in traditional medicine for relieving alcohol intoxication.
T17	Used in traditional medicine for relieving cough.
T18	Used in traditional medicine for relieving depression.
T19	Used in traditional medicine for relieving dryness.
T20	Used in traditional medicine for relieving pain.
T21	Used in traditional medicine for relieving summer heat.
T22	Used in traditional medicine for removing dampness.
T23	Used in traditional medicine for stopping bleeding.
T24	Used in traditional medicine for treating childhood malnutrition.
T25	Used in traditional medicine for treating cold.
T26	Used in traditional medicine for treating diuresis.
T27	Used in traditional medicine for treating fever.
T28	Used in traditional medicine for treating physical weakness.
T29	Used in traditional medicine for treating rheumatic pain.
T30	Used in traditional medicine for treating sore throat.
T31	Used in traditional medicine for treating stomach disorders.
T32	Used in traditional medicine for treating toothache.
T33	Used in traditional medicine for treating wind-cold.
T34	Used in traditional medicine for treating wind-heat syndrome and common cold.
T35	Used in traditional medicine for treating lung-heat cough.
T36	Used in traditional medicine for warming the stomach.

https://doi.org/10.3390/biology15110873
